# Supplementary material for: Gene Mapping, Genome-Wide Transcriptome Analysis, and WGCNA Reveals the Molecular Mechanism for Triggering Programmed Cell Death in Rice Mutant pir1
Source: Plants (Basel). 2020 Nov 19;9(11):1607. doi: 10.3390/plants9111607 (PMC7699392; doi:10.3390/plants9111607)
Supplement: Supplementary file 1 [file plants-09-01607-s001.zip › Supplementary files/Figure S1-S11.docx]

Gene Mapping, Genome-Wide Transcriptome Analysis and WGCNA Reveals the Molecular Mechanism for Triggering Programmed Cell Death in Rice Mutant *pir1*

Xinyu Chen ^1,†^, Qiong Mei ^2,3, †^, Weifang Liang ^4,†^, Jia Sun ^5^, Xuming Wang ^2^, Jie Zhou ^2^, Junmin Wang ^2^, Yuhang Zhou ^1^, Bingsong Zheng ^1^, Yong Yang ^2,*^ and Jianping Chen ^2,6,*^

Supplementary Materials


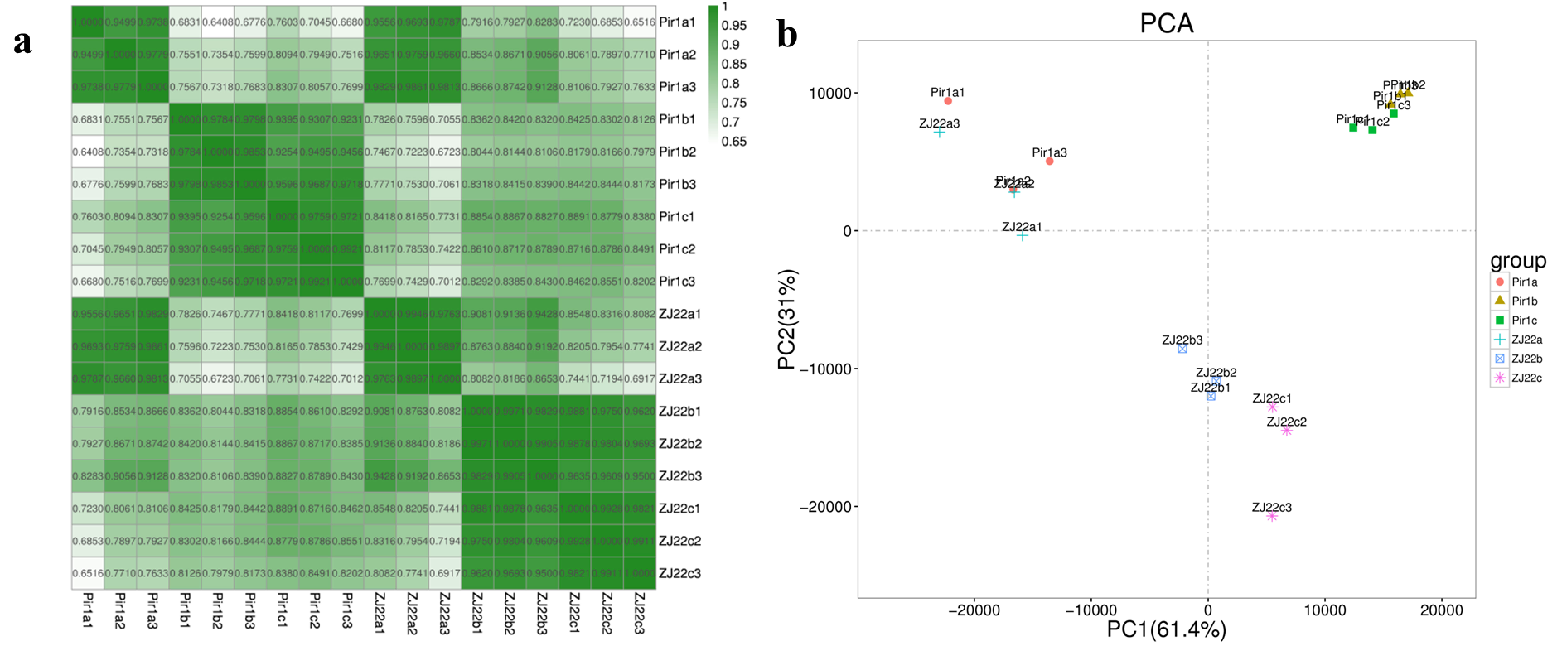


**Figure S1.** Correlation analysis among the samples including ZJ22a, ZJ22b, ZJ22c, *pir1*a, *pir1*b and *pir1*c. (a) The correlation coefficient between each pair of samples showing the biological repeatability. The color intensity represents the p-value. (b) Principal component analysis (PCA) showing the correlation among samples at the level of two PCs.


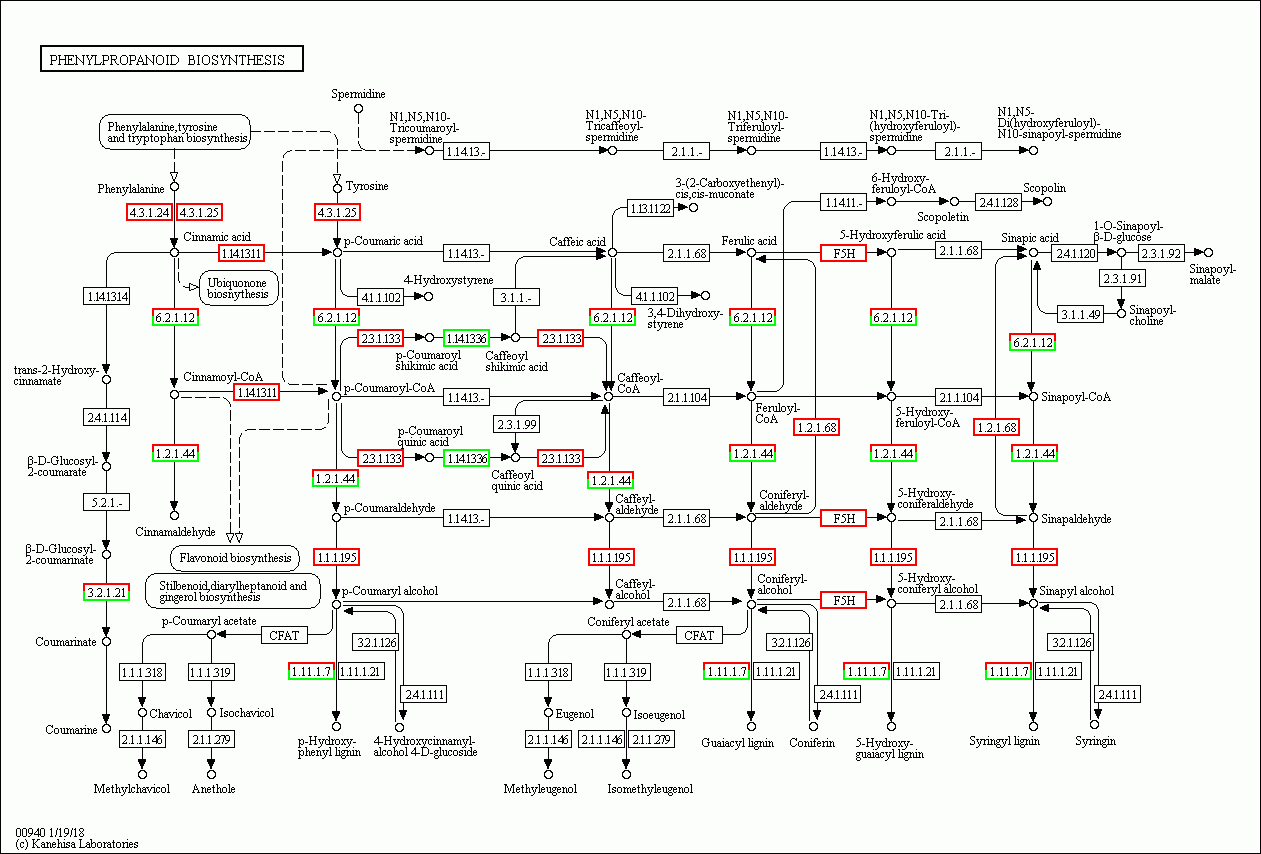


a


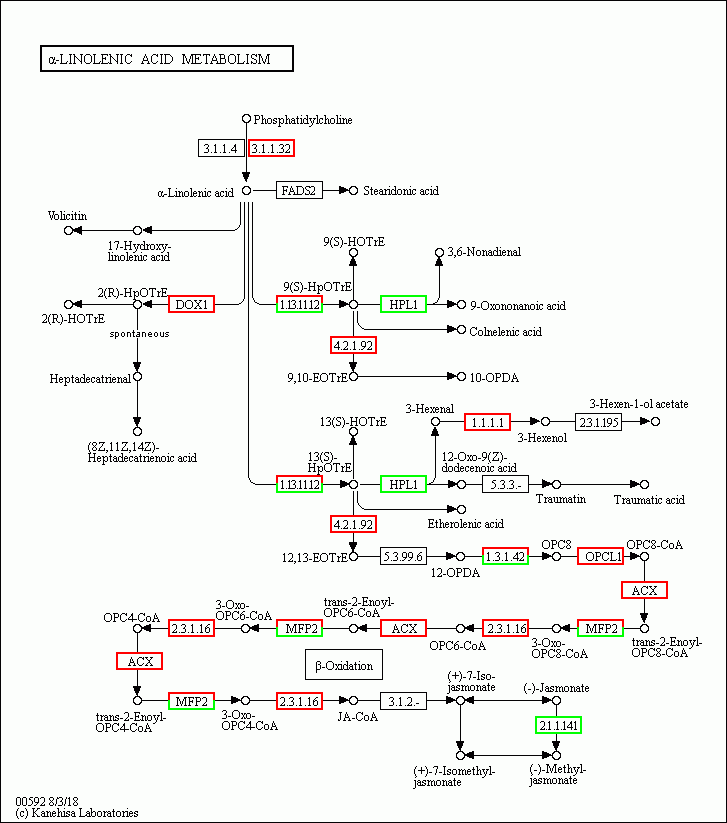


b


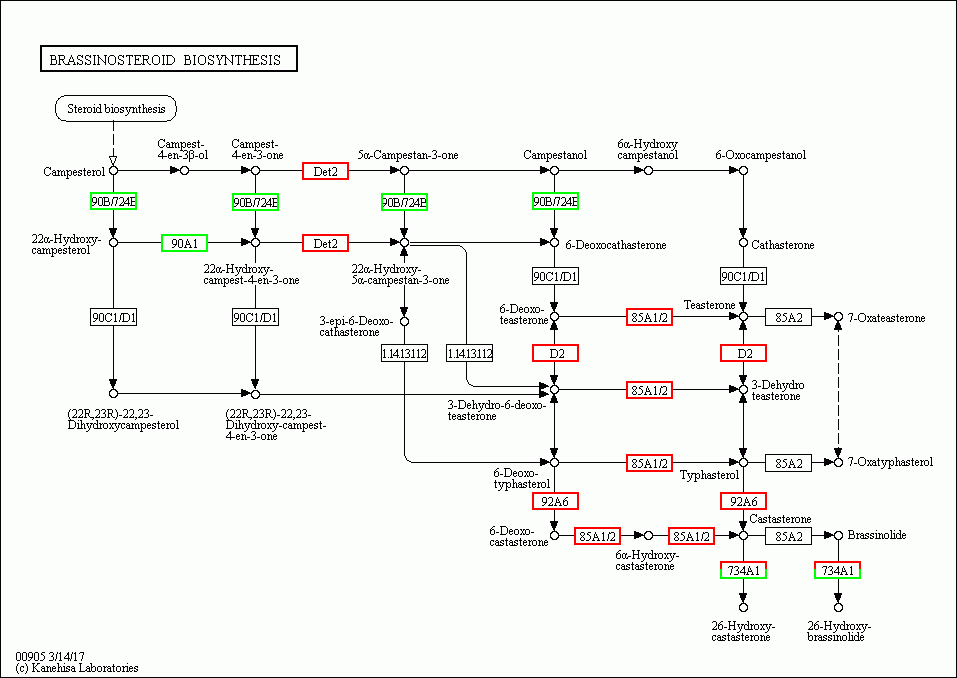


c

**Figure S2**. The pathways of phenylpropanoid biosynthesis (a); alpha-linolenic acid metabolism (b); and brassinosteroid biosynthesis (c) enriched by KEGG analysis. The KEGG pathway database is gained from Kanehisa laboratories.


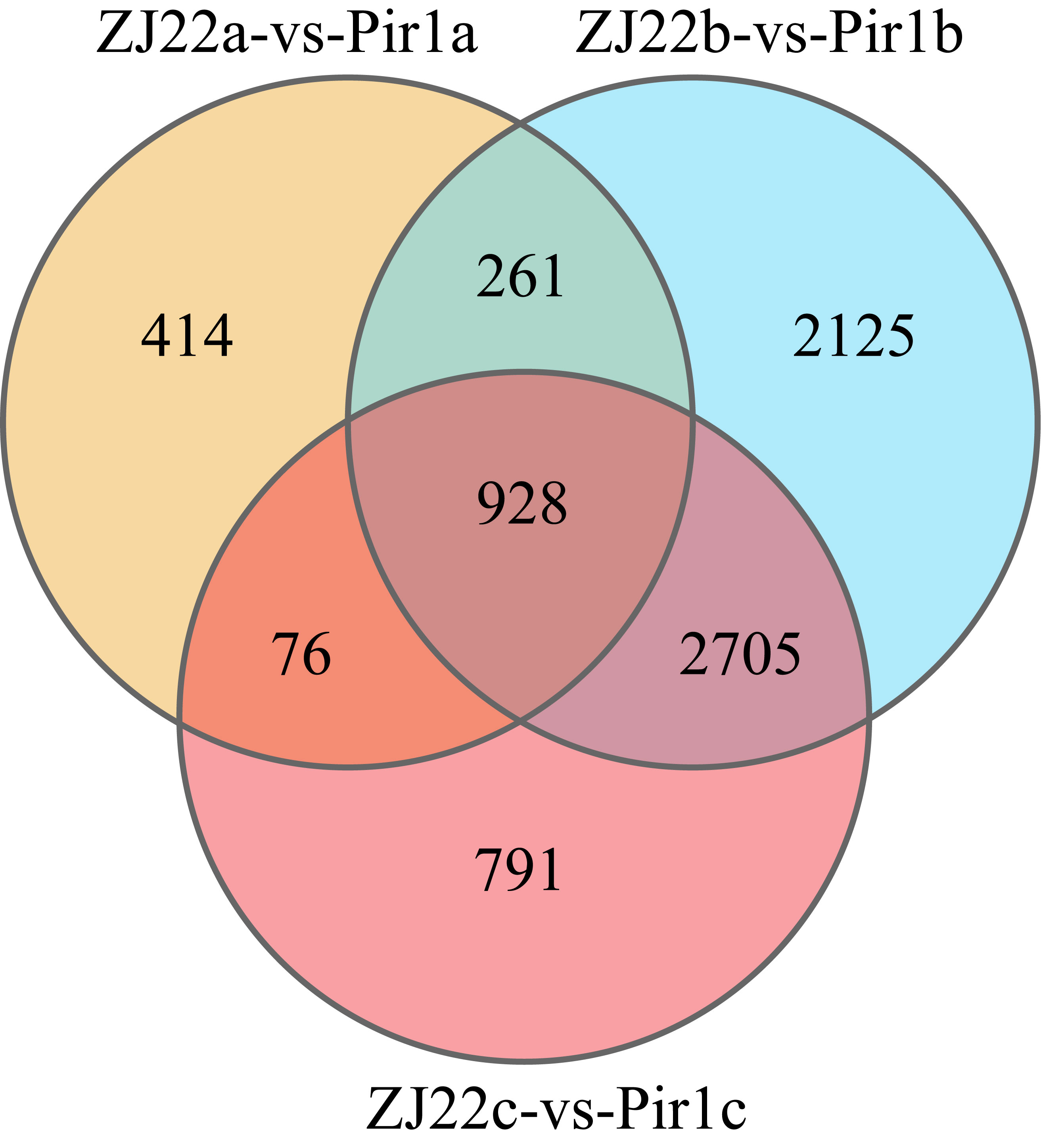

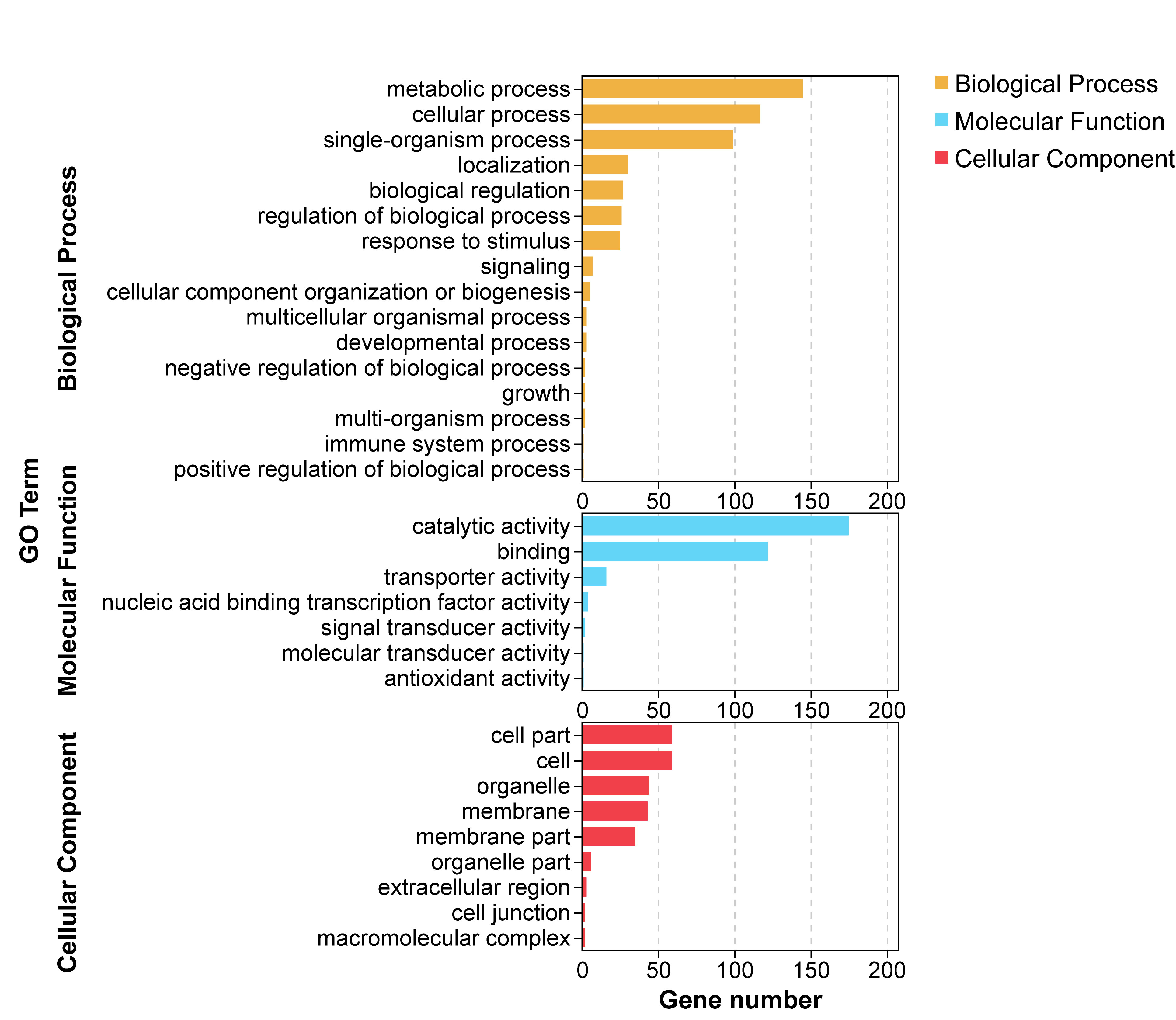

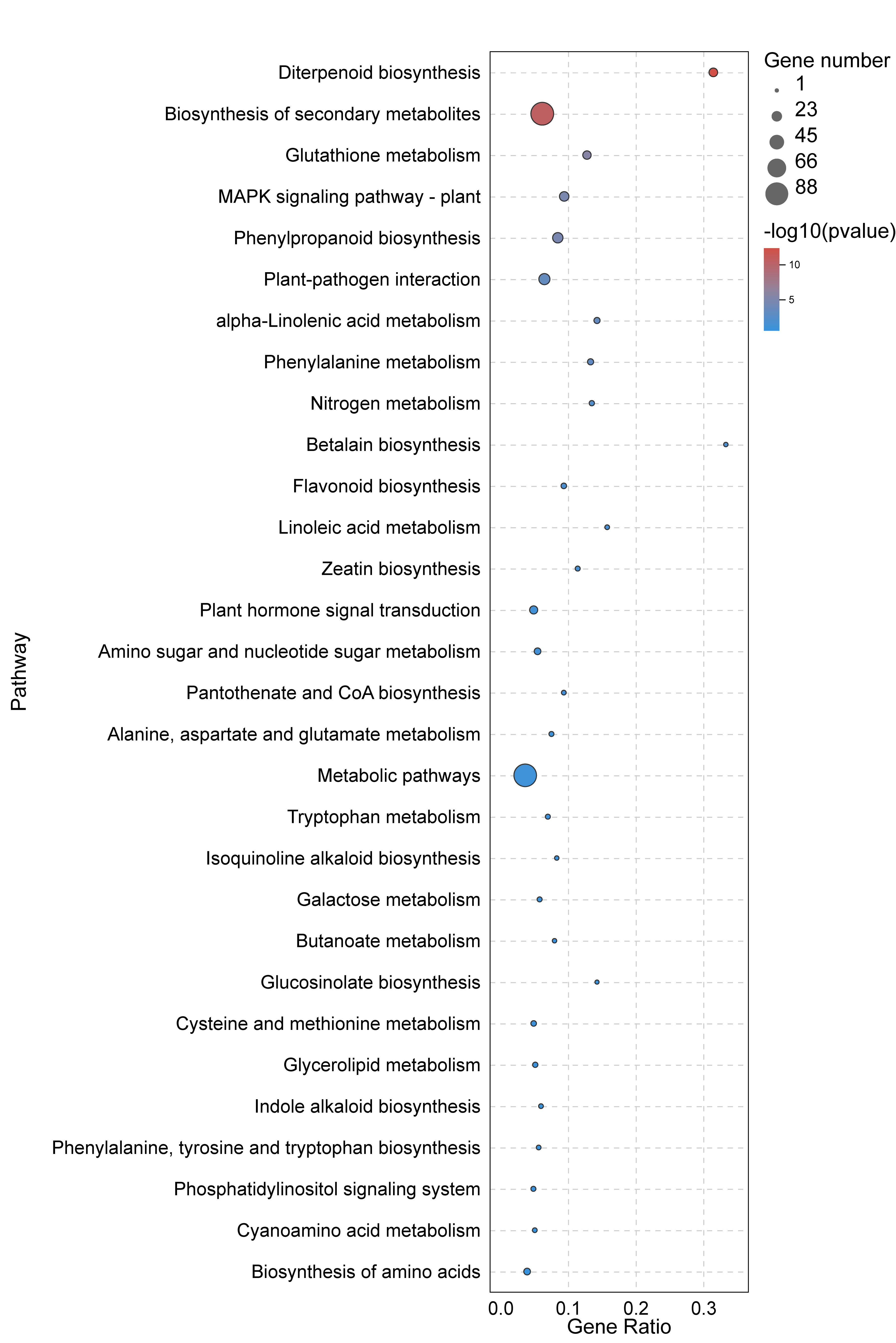


a

b

**Figure S3**. Common DEGs analysis in wild-type and mutant comparison. (a) Venn diagram of the number of DEGs in wild-type and mutant comparison. ZJ22a vs. *pir1*a, ZJ22b vs. *pir1*b, ZJ22c vs. *pir1*c; (b) GO terms enrichment for the 928 common genes; (c) Top 30 enriched KEGG pathways.

c


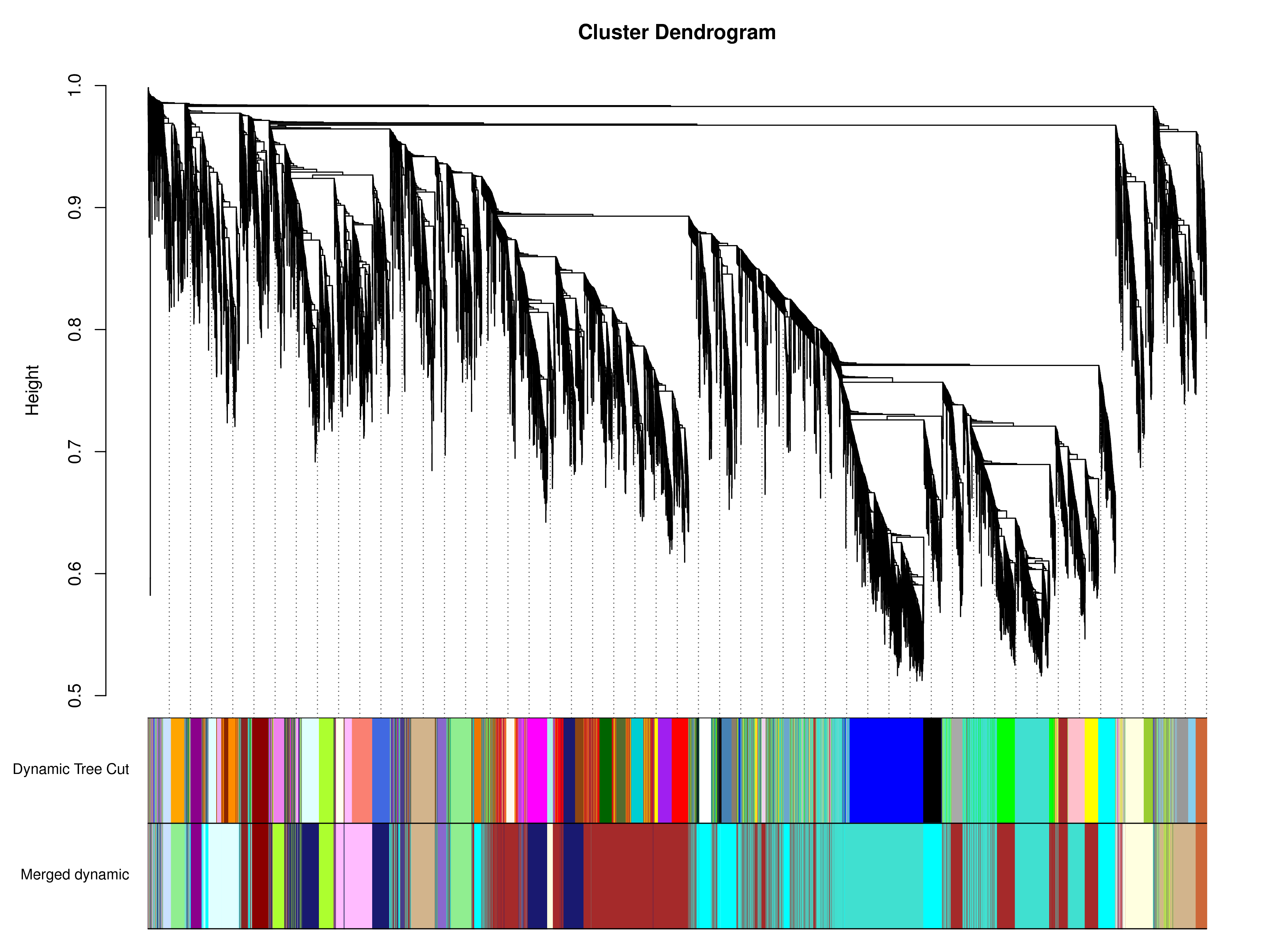


a


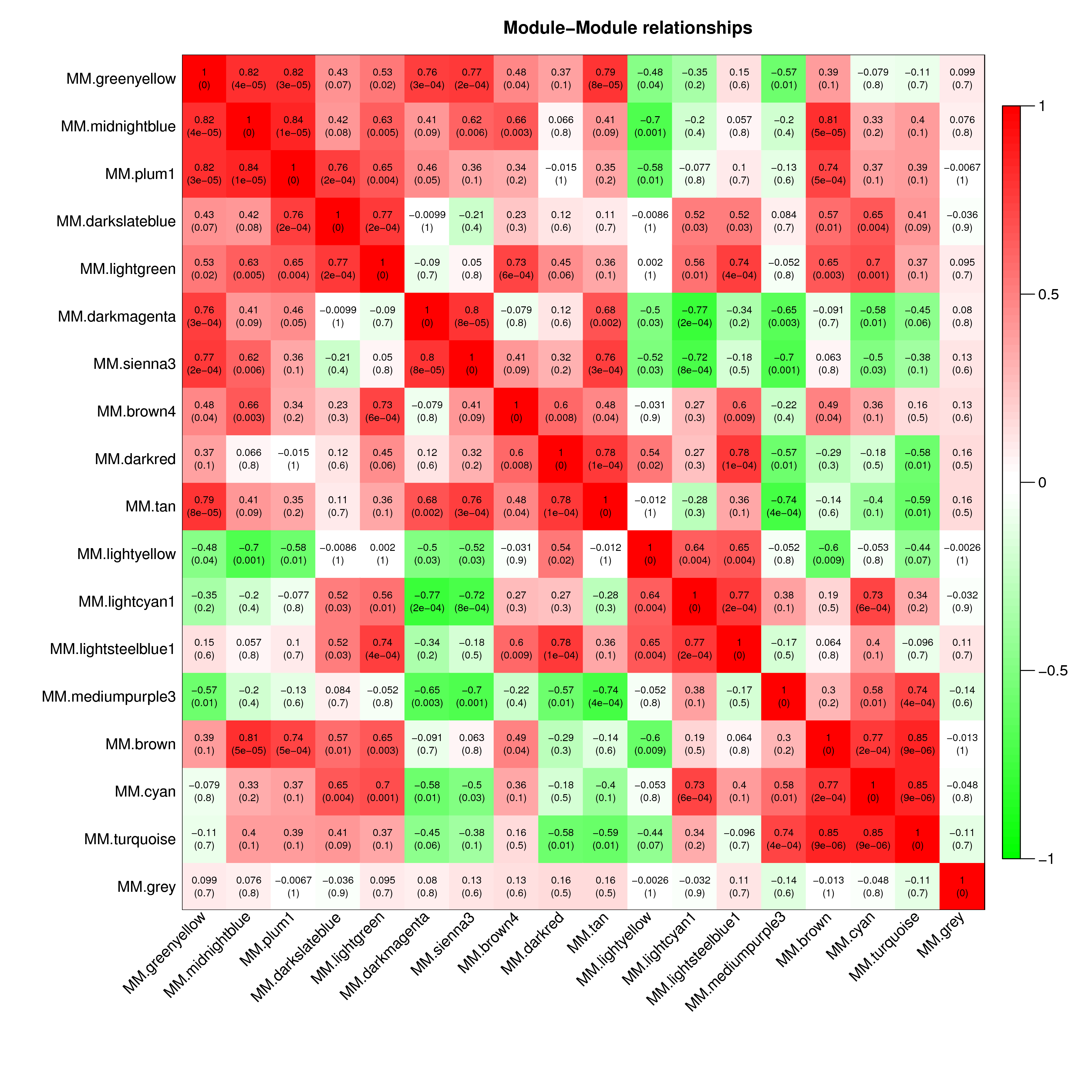


b

**Figure S4**. Network analysis dendrogram showing modules identified by weighted gene co-expression network analysis (WGCNA). (a) Dendrogram plot with color annotation. (b) Module-module membership in WGCNA and corresponding P-values. The upper panel shows the 17 modules labeled with different colors. The color scale on the right shows module-trait correlation from −1 (green) to 1 (red)


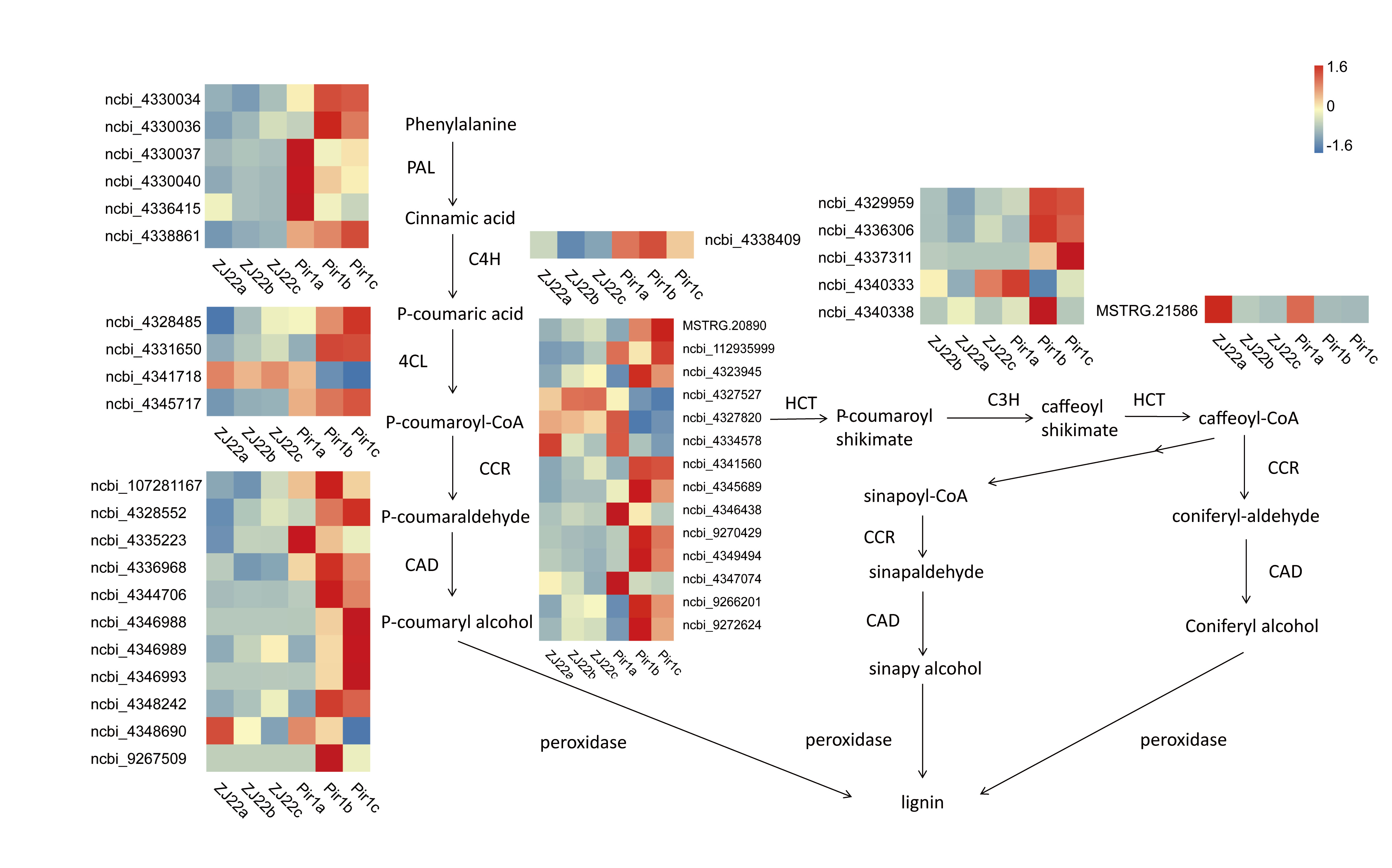


**Figure S5**. Heatmaps of expression patterns for the DEGs involved in lignin biosynthesis.


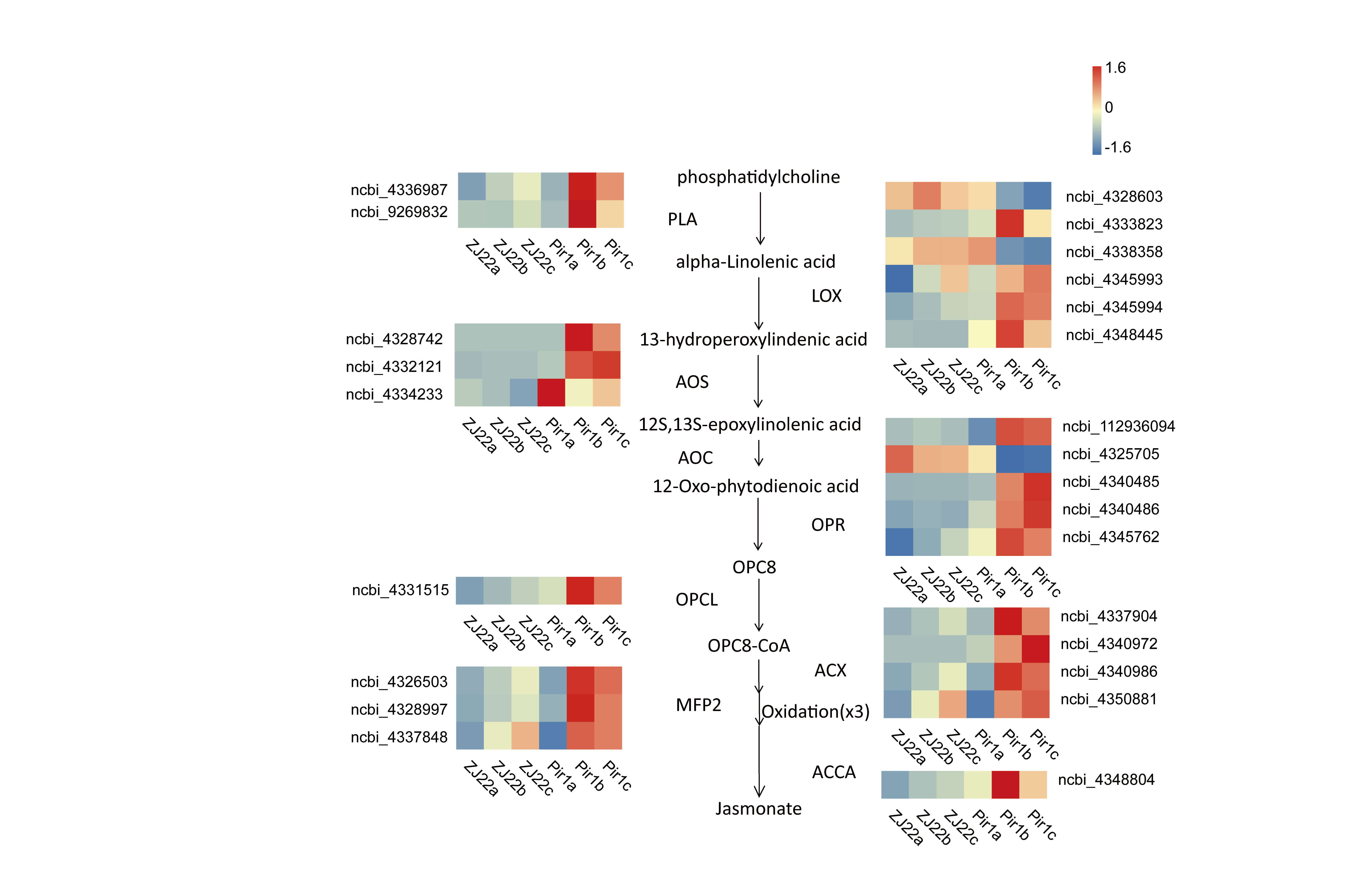


**Figure S6**. Heatmaps of expression patterns for DEGs involved in JA biosynthesis.


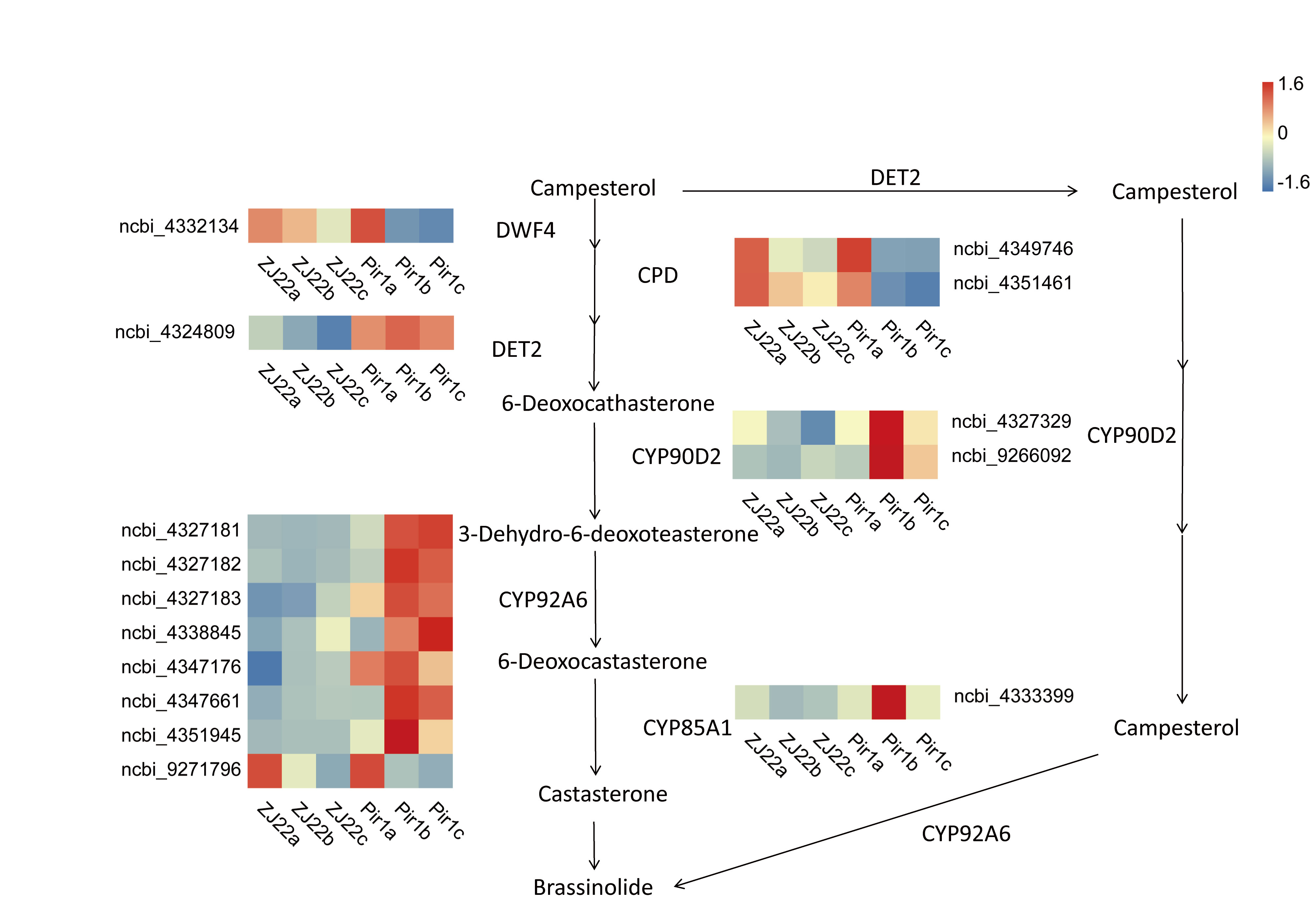


**Figure S7**. Heatmaps of expression patterns for DEGs involved in BR biosynthesis.


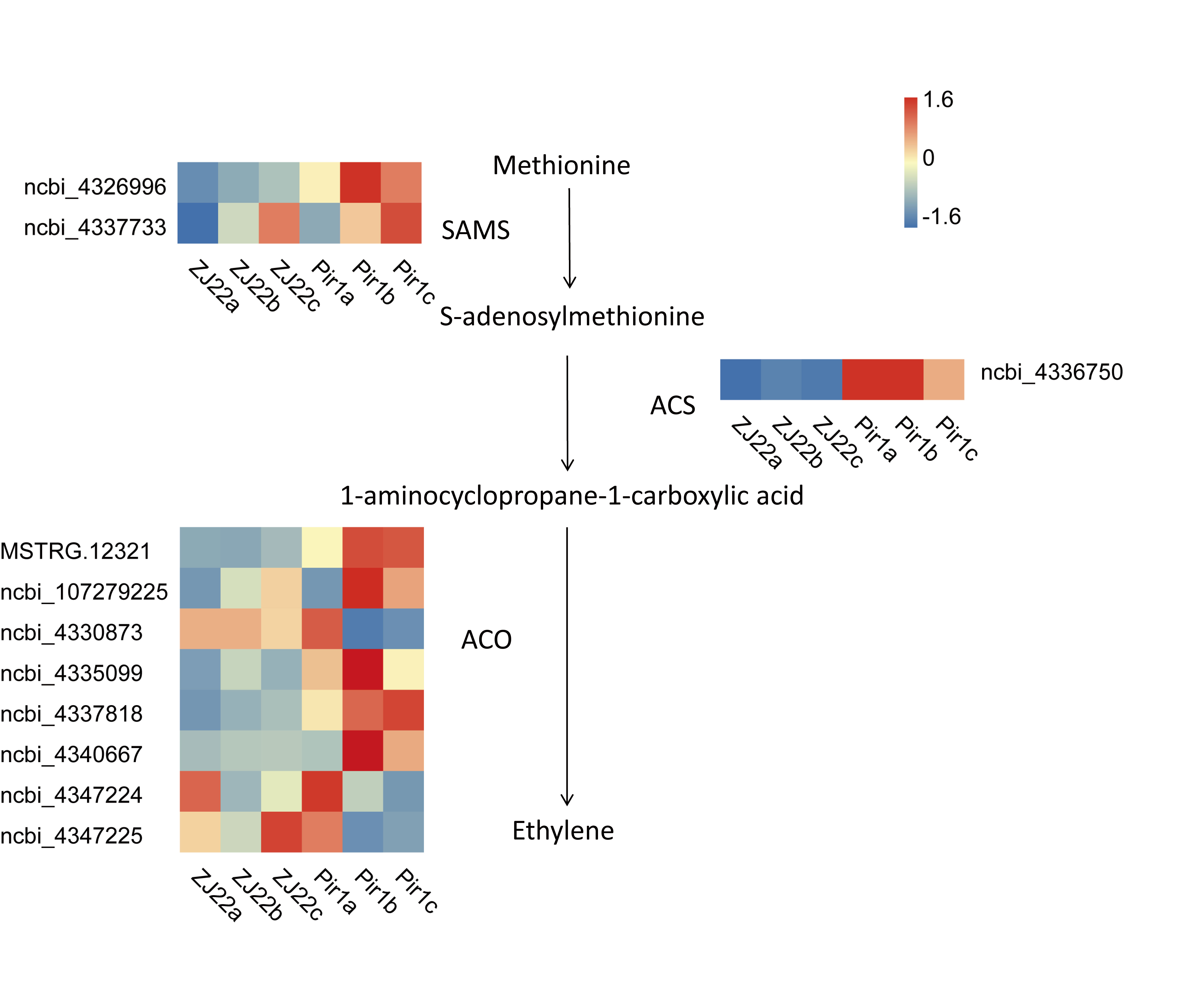


**Figure S8**. Heatmaps of expression patterns for DEGs involved in ET biosynthesis.


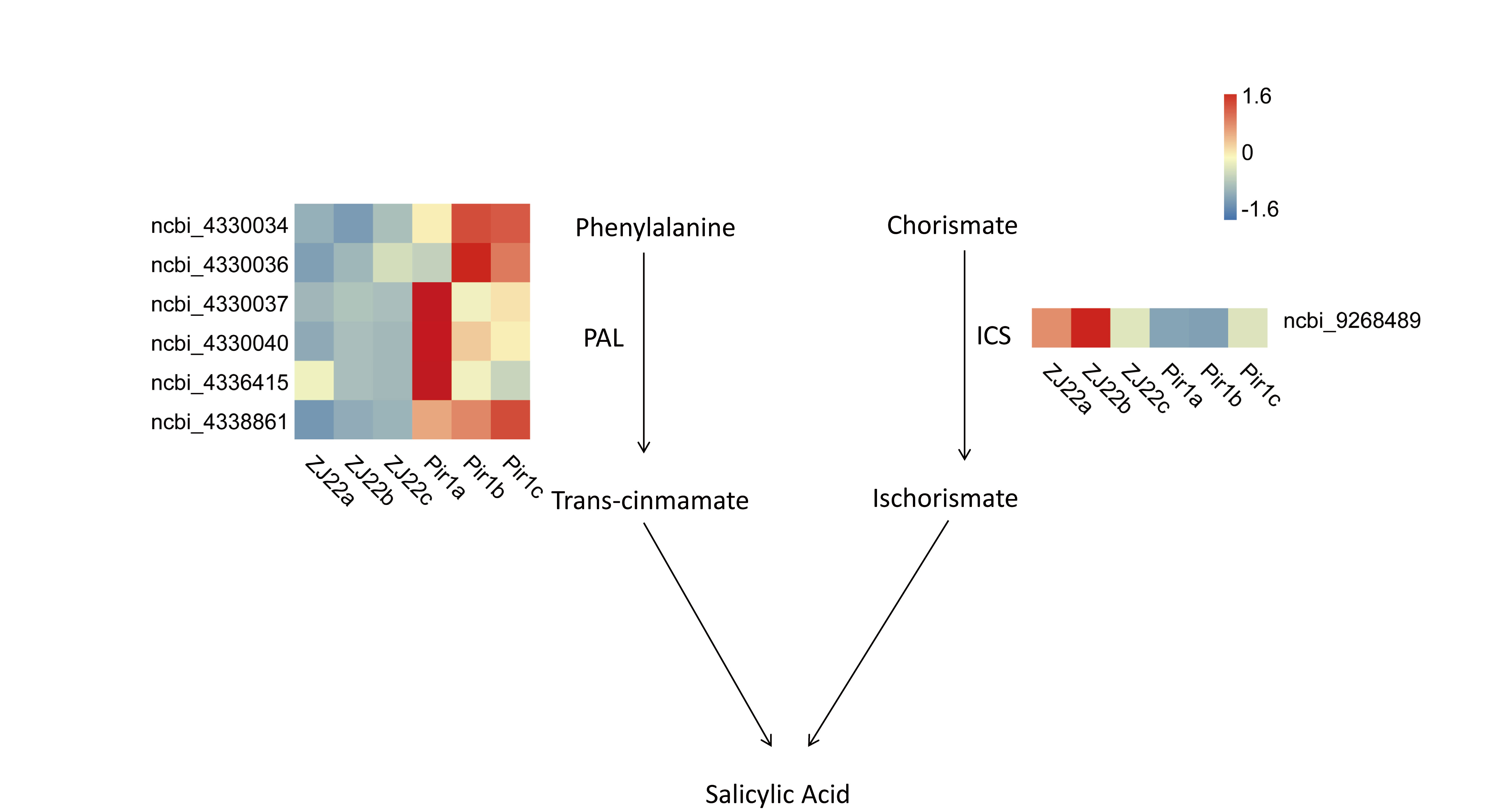


**Figure S9**. Heatmaps of expression patterns for DEGs involved in SA biosynthesis


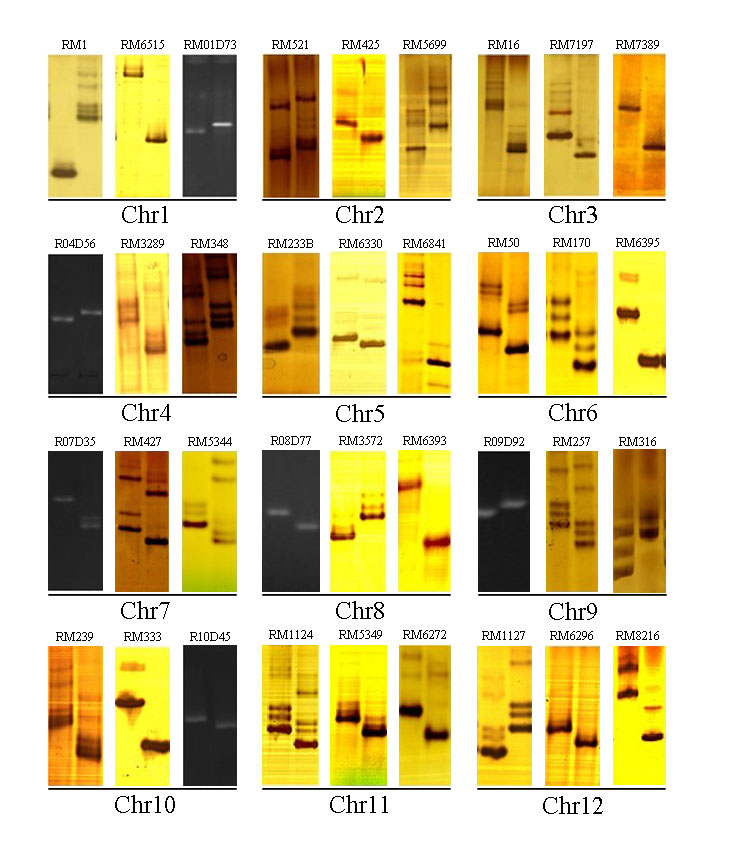


**Figure S10**. Electrophoretogram of molecular markers with polymorphism. Three markers randomly selected on each chromosome for illustration, left: *pir1*; right: 9311.


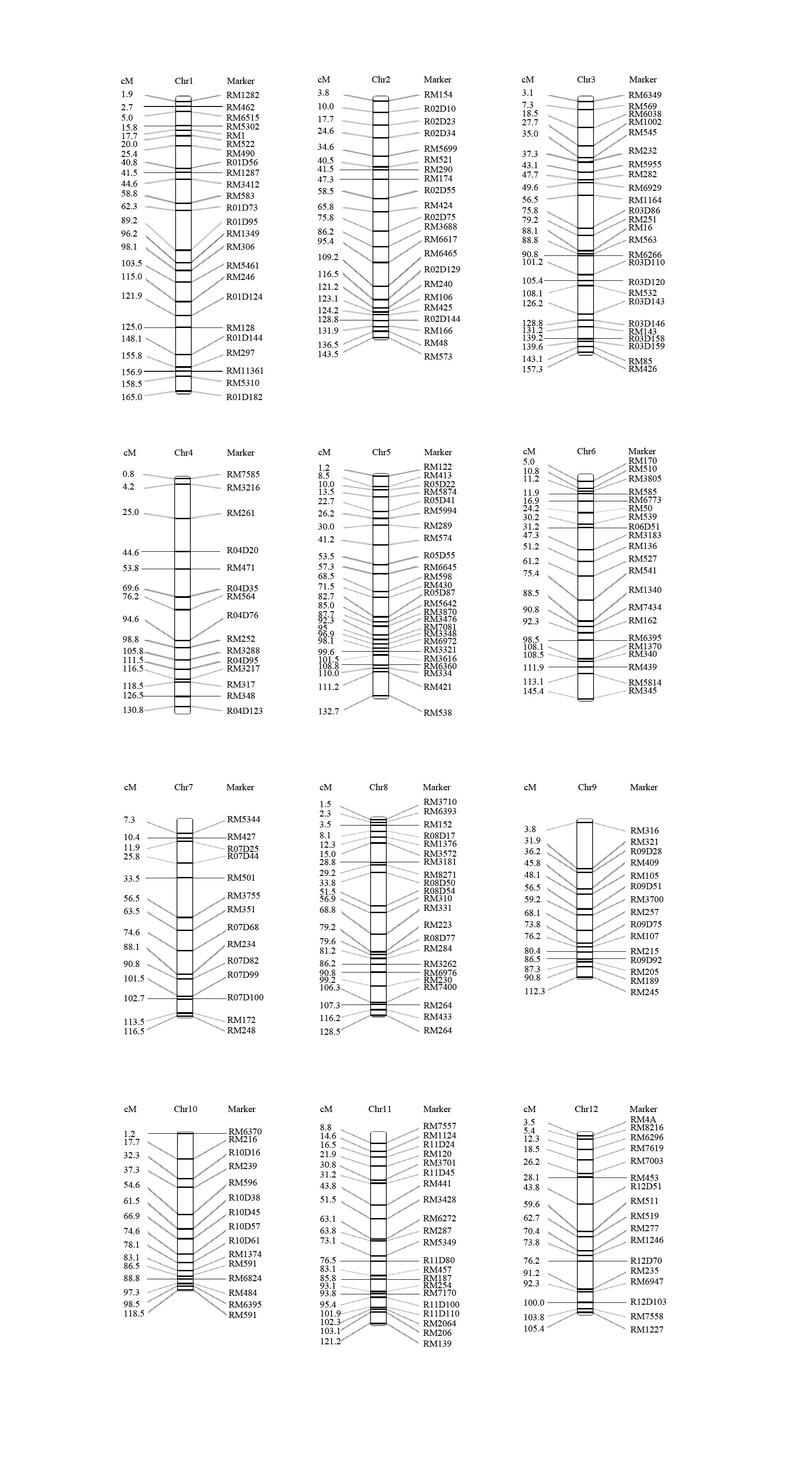


**Figure S11.** Genetic linkage map of the polymorphic markers. Distances (cM) of markers on chromosome and marker names are shown on the left and right sides of the chromosomes, respectively.
