# Supplementary material for: Gene Mapping, Genome-Wide Transcriptome Analysis, and WGCNA Reveals the Molecular Mechanism for Triggering Programmed Cell Death in Rice Mutant pir1
Source: Plants (Basel). 2020 Nov 19;9(11):1607. doi: 10.3390/plants9111607 (PMC7699392; doi:10.3390/plants9111607)
Supplement: Supplementary file 1 [file plants-09-01607-s001.zip › Supplementary files/Table S1.docx]

**Table S1**. Genetic analysis of F_2_ populations

| Cross | Total number of plants | Number of normal plants | Number of plants  with lesion mimic | χ^2^(3:1) | χ^2^_0.05_ |
| --- | --- | --- | --- | --- | --- |
| ZJ22 × *pir1* | 131 | 94 | 37 | 0.57 | 3.84 |
| 9311 × *pir1* | 109 | 74 | 35 | 2.57 | 3.84 |
