# Supplementary material for: Gene Mapping, Genome-Wide Transcriptome Analysis, and WGCNA Reveals the Molecular Mechanism for Triggering Programmed Cell Death in Rice Mutant pir1
Source: Plants (Basel). 2020 Nov 19;9(11):1607. doi: 10.3390/plants9111607 (PMC7699392; doi:10.3390/plants9111607)
Supplement: Supplementary file 1 [file plants-09-01607-s001.zip › Supplementary files/Table S33.docx]

**Table S33.** The DEGs encoding proteins involved in MAPK cascades, calcium signaling, NADPH oxidase and antioxidative metabolism

| **Group** | **Gene ID** | **Log_2_(*pir1*/ZJ22)** | | | **Description** |
| --- | --- | --- | --- | --- | --- |
|  |  | **a** | **b** | **c** |  |
| MAPK cascades | LOC4340367 | -0.50 | -0.79 | -0.89 | mitogen-activated protein kinase kinase 5 |
|  | LOC4328223 | 2.44 | 1.66 | 1.39 | mitogen-activated protein kinase 13 isoform X1 |
|  | LOC9271851 | 3.30 | 2.25 | 0.87 | mitogen-activated protein kinase kinase kinase 1 isoform X1 |
|  | LOC4332475 | 2.08 | 1.68 | 1.62 | mitogen-activated protein kinase 5 isoform X1 |
|  | LOC4328297 | 0.42 | 1.67 | 0.66 | mitogen-activated protein kinase 3 |
|  | LOC4344698 | -0.42 | -1.03 | -0.87 | mitogen-activated protein kinase 2 |
|  | LOC4342017 | 0.22 | 0.15 | -0.28 | mitogen-activated protein kinase 12 |
|  | LOC4339697 | 0.78 | 0.42 | 1.20 | mitogen-activated protein kinase 17 isoform X1 |
|  | LOC4326567 | 0.72 | 0.79 | 0.53 | mitogen-activated protein kinase 16 |
|  | LOC4339497 | 1.19 | 2.54 | 0.83 | mitogen-activated protein kinase kinase kinase 2 |
|  | LOC4332115 | 0.33 | 0.19 | 0.35 | mitogen-activated protein kinase kinase 9 |
|  | LOC4340126 | 0.59 | -0.05 | -0.12 | mitogen-activated protein kinase kinase 2 |
|  | LOC4330859 | 0.11 | 0.14 | 0.3 | mitogen-activated protein kinase kinase kinase 1 |
|  | LOC4333845 | 0.04 | 0.46 | 0.28 | mitogen-activated protein kinase kinase kinase 1 |
|  | LOC4340368 |  | 7.09 | 6.20 | mitogen-activated protein kinase kinase 5 |
|  | LOC4341956 | 0.46 | 0.25 | 0.27 | mitogen-activated protein kinase 4 |
|  | LOC9267453 | -0.10 | 0.72 | -0.04 | mitogen-activated protein kinase kinase 3 isoform X1 |
|  | LOC4325301 | 0.29 | 2.36 | 0.87 | mitogen-activated protein kinase kinase kinase 17 |
|  | LOC4329172 | -0.34 | 1.18 | 1.09 | mitogen-activated protein kinase kinase kinase NPK1 |
|  | LOC4340170 | 0.29 | 0.52 | -0.02 | mitogen-activated protein kinase 1 |
|  | LOC4330957 | 0.90 | -0.51 | -0.02 | mitogen-activated protein kinase kinase 5 |
|  | LOC107280961 | 2.32 | 3.32 | 3.74 | mitogen-activated protein kinase kinase 5-like |
|  | LOC4339496 | -1.18 | 1.76 | 0.74 | mitogen-activated protein kinase kinase kinase ANP1 |
|  | LOC4332325 | 0.37 | 0.23 | 0.02 | mitogen-activated protein kinase kinase kinase 1 |
|  | LOC107275541 | 4.91 |  | 3.74 | mitogen-activated protein kinase kinase 9-like |
|  | LOC107277074 |  | 5.64 |  | mitogen-activated protein kinase kinase 9-like |
| calcium signaling | LOC4349412 | 0.44 | 0.84 | 0.24 | calcineurin B-like protein 1 |
|  | LOC4325933 | -0.11 | 1.43 | 1.24 | calcineurin B-like |
|  | LOC4352701 | 0.03 | 1.16 | 0.76 | calcineurin B-like protein 2 |
|  | LOC4333502 | -0.16 | 0.73 | 0.48 | calcineurin B-like protein 3 |
|  | LOC107276218 |  | -4.26 |  | calcineurin B-like protein 3 |
|  | LOC4339432 | 0.33 | -0.58 | 0.44 | calcineurin B-like protein 4 isoform X1 |
|  | LOC4325962 | 2.09 | 4.03 | 3.78 | calcineurin B-like protein 5 isoform X1 |
|  | LOC4351575 | -0.08 | 0.21 | 0.29 | calcineurin B-like protein 6 |
|  | LOC107276277 | 1.42 | 1.16 | 2.97 | calcineurin B-like protein 7 |
|  | LOC9269084 | -1.49 | -1.67 | -2.53 | calcineurin B-like protein 9 |
|  | LOC4332162 | 2.96 | 1.51 | 5.28 | calcium uniporter protein 4, mitochondria |
|  | LOC4349038 | 0.92 | 1.48 | 0.25 | calcium uniporter protein 2, mitochondrial |
|  | LOC4349639 | 0.65 | 0.93 | 0.20 | calcium uniporter protein 6, mitochondrial |
|  | LOC4351363 | 0.49 | 0.29 | 0.08 | calcium uniporter protein 6, mitochondrial |
|  | LOC4327489 | 0.41 | 0.75 | 0.78 | calcium uniporter protein 6, mitochondrial |
|  | LOC112936018 | 0.51 | 0.01 | -0.79 | calcium-dependent protein kinase 16 isoform X2 |
|  | LOC4326638 | 0.19 | 0.32 | 0.22 | calcium-dependent protein kinase 3 |
|  | LOC4327438 | 0.14 | -0.46 | -0.29 | calcium-dependent protein kinase 13 |
|  | LOC4327560 | 0.56 | -4.74 | -3.74 | calcium-dependent protein kinase 34 |
|  | LOC4328155 | 0.68 | -0.36 | -0.04 | calcium-dependent protein kinase 16 isoform X1 |
|  | LOC4330351 | -0.20 | -0.55 | -0.44 | calcium-dependent protein kinase 26 |
|  | LOC4331257 | -0.37 | -0.65 | -1.15 | calcium-dependent protein kinase 26 |
|  | LOC4331490 | -0.70 | -1.22 | -1.28 | calcium-dependent protein kinase isoform 11 |
|  | LOC4333767 | -0.42 | 2.00 | 1.42 | calcium-dependent protein kinase 10 |
|  | LOC4334371 | -1.89 | -0.39 | 0.24 | calcium-dependent protein kinase 26 |
|  | LOC4334519 | 0.26 | 1.33 | 1.09 | calcium-dependent protein kinase 8 |
|  | LOC4336508 | -0.12 | 0.33 | -0.05 | calcium-dependent protein kinase 27-like |
|  | LOC4336653 | -0.57 | -2.57 | -1.94 | calcium-dependent protein kinase 2 |
|  | LOC4336783 | 0.91 | 0.60 | 0.37 | calcium-dependent protein kinase 26 |
|  | LOC4339177 | 2.86 | -1.70 | -2.52 | calcium-dependent protein kinase 34 |
|  | LOC4339751 | 0.44 | 0.74 | 0.56 | calcium-dependent protein kinase 3 |
|  | LOC4339976 | -0.11 | 0.26 | 0.32 | calcium-dependent protein kinase 26 |
|  | LOC4342456 | 0.09 | 0.20 | 0.31 | calcium-dependent protein kinase 26 |
|  | LOC4343002 | 0.61 | 0.84 | 0.45 | calcium-dependent protein kinase 28 |
|  | LOC4343373 | -0.08 | -0.39 | -0.46 | calcium-dependent protein kinase isoform 2 |
|  | LOC4343644 | 0.24 | 0.51 | 0.53 | calcium-dependent protein kinase 8 |
|  | LOC4346187 | 0.19 | -0.61 | 0.28 | calcium-dependent protein kinase 13 |
|  | LOC4347502 | -0.06 | 0.45 | 0.13 | calcium-dependent protein kinase 14 |
|  | LOC4347564 | -0.63 | -0.17 | 0.32 | calcium-dependent protein kinase 32 |
|  | LOC4349249 | 0.34 | -1.05 | -0.97 | calcium-dependent protein kinase isoform 1 |
|  | LOC4349714 | -0.74 | 0.94 | 0.85 | calcium-dependent protein kinase 34 |
|  | LOC4349899 | 0.25 | 0.07 | -0.10 | calcium-dependent protein kinase SK5 |
|  | LOC4351620 | -0.18 | 0.03 | -0.04 | calcium-dependent protein kinase 11 |
|  | LOC4351829 |  | -1.26 | -1.36 | calcium-dependent protein kinase 7 |
|  | LOC4324384 | 0.43 | 1.10 | 0.70 | calmodulin-7-like |
|  | LOC4332664 | 0.23 | -0.26 | -0.40 | calmodulin-7-like |
|  | LOC4339172 | -0.10 | 0.37 | 0.29 | calmodulin-2 |
|  | LOC4326903 | 0.47 | 1.63 | 0.94 | calmodulin-3 |
| NADPH oxidase | LOC4345742 | 1.42 | 1.12 | 0.13 | respiratory burst oxidase homolog protein E |
|  | LOC4327453 | 0.82 | -2.12 | -2.48 | respiratory burst oxidase1 |
|  | LOC4347158 | 0.67 | 0.49 | -0.48 | respiratory burst oxidase homolog protein E |
|  | LOC107278041 | 0.30 | 1.62 | 1.45 | respiratory burst oxidase homolog protein B-like |
|  | LOC4324163 | 0.32 | 0.52 | 0.43 | respiratory burst oxidase homolog protein A |
|  | LOC4339397 | 0.27 | -0.26 | -0.50 | respiratory burst oxidase homolog protein A |
|  | LOC4326027 | 0.24 | -2.10 | -2.46 | respiratory burst oxidase homolog protein B |
| POD | LOC4347962 | 1.19 | -5.94 | -5.63 | peroxidase A2 isoform X1 |
|  | LOC4332174 | 0.41 |  |  | peroxidase A2 |
| CAT | LOC4342124 | -0.48 | 1.48 | 0.96 | catalase isozyme B |
|  | LOC4328073 | 0.62 | 2.29 | 1.27 | catalase isozyme A |
|  | LOC4331509 | -0.40 | -1.08 | -0.88 | catalase-1 |
| SOD | LOC4332846 | 0.65 | 1.07 | 0.87 | superoxide dismutase [Cu-Zn] 1 |
|  | LOC4336726 | 0.54 | 0.82 | 0.90 | copper chaperone for superoxide dismutase, chloroplastic |
|  | LOC4334586 | -0.69 | 0.71 | -0.69 | copper chaperone for superoxide dismutase, chloroplastic-like |
|  | LOC4346329 | 0.21 | 0.34 | 0.58 | superoxide dismutase [Cu-Zn], chloroplastic |
|  | LOC4332082 | -0.14 | 0.12 | -0.17 | superoxide dismutase [Cu-Zn] 2 isoform X1 |
|  | LOC4340091 | -0.32 | -0.25 | -0.19 | superoxide dismutase [Fe] 2, chloroplastic |
|  | LOC4338417 | -0.14 | 0.32 | 0.16 | superoxide dismutase [Mn], mitochondrial |
|  | LOC4339922 | -0.17 | -0.15 | 0.035 | superoxide dismutase [Fe] 1, chloroplastic |
| GR | LOC4331112 | 0.17 | 1.31 | 0.88 | glutathione reductase, cytosolic |
|  | LOC4331719 | -0.35 | -0.25 | 0.17 | glutathione reductase, chloroplastic |
|  | LOC4348623 | 0.12 | 1.53 | 1.36 | glutathione reductase, chloroplastic |
| APX | LOC4335896 | 1.48 | 3.24 | 2.50 | probable L-ascorbate peroxidase 7, chloroplastic |
|  | LOC4329643 | -0.31 | -1.51 | -0.83 | probable L-ascorbate peroxidase 8, chloroplastic isoform X1 |
|  | LOC9269342 | 0.45 | 1.28 | 0.85 | probable L-ascorbate peroxidase 4 |
|  | LOC4344397 | -0.02 | 0.63 | 0.74 | L-ascorbate peroxidase 2, cytosolic |
|  | LOC4351663 | -0.27 | 0.44 | 0.56 | probable L-ascorbate peroxidase 6, chloroplastic |
|  | LOC4351664 | -0.32 | -0.52 | -0.26 | probable L-ascorbate peroxidase 5, chloroplastic isoform X1 |
|  | LOC4335202 | 1.85 | 1.34 | 2.78 | probable L-ascorbate peroxidase 3 |
|  | LOC4346247 | 0.03 | -0.18 | -0.04 | probable L-ascorbate peroxidase 4 |
|  | LOC4332474 | 0.15 | 0.33 | 0.10 | L-ascorbate peroxidase 1, cytosolic |
