# Supplementary material for: Gene Mapping, Genome-Wide Transcriptome Analysis, and WGCNA Reveals the Molecular Mechanism for Triggering Programmed Cell Death in Rice Mutant pir1
Source: Plants (Basel). 2020 Nov 19;9(11):1607. doi: 10.3390/plants9111607 (PMC7699392; doi:10.3390/plants9111607)
Supplement: Supplementary file 1 [file plants-09-01607-s001.zip › Supplementary files/Table S34.docx]

**Table S34.** Molecular markers with polymorphism between *pir1* and 9311.

| **Marker** | **Chromosome** | **Position (Mb)** | **Type** | **Forward Primer** | **Reverse Primer** |
| --- | --- | --- | --- | --- | --- |
| RM1282 | 1 | 0.5 | SSR | AAGCATGACAGCTGCAAGAC | GGGGATGAAGGGTAATTTCG |
| RM462 | 1 | 0.7 | SSR | ACGGCCCATATAAAAGCCTC | AAGATGGCGGAGTAGCTCAG |
| RM6515 | 1 | 1.3 | SSR | CTCGGCTAGTGACGATTTCTTGG | ACGTCGTGGTAGGCGACATAGC |
| RM5302 | 1 | 4.1 | SSR | TATGGGTGACACATTGGGAC | TTGTGACGTTTGAGAGCTGG |
| RM1 | 1 | 4.6 | SSR | GCGAAAACACAATGCAAAAA | GCGTTGGTTGGACCTGAC |
| RM522 | 1 | 5.2 | SSR | ACCAGAGAAGCCCTCCTAGC | GTTCTGTGGTGGTCACGTTG |
| RM490 | 1 | 6.6 | SSR | ATCTGCACACTGCAAACACC | AGCAAGCAGTGCTTTCAGAG |
| R01D56 | 1 | 10.6 | STS | ATTCCTGGTTCTACATTACTTA | CGCCTCACTAGAATATCGGA |
| RM1287 | 1 | 10.8 | SSR | CCATTTGCAGTATGAACCATGC | ATCATGCAATAGCCGGTAGAGG |
| RM3412 | 1 | 11.6 | SSR | TGATGGATCTCTGAGGTGTAAAGAGC | TGCACTAATCTTTCTGCCACAGC |
| RM583 | 1 | 15.3 | SSR | AGATCCATCCCTGTGGAGAG | GCGAACTCGCGTTGTAATC |
| R01D73 | 1 | 16.2 | STS | CTTCCTTGCTGGAGAGCTG | TTATGATGGTGCCTCCCATT |
| R01D95 | 1 | 23.2 | STS | GTAAAGCATGCGTGCACACC | TTAATAAGAGGCAACGGAAC |
| RM1349 | 1 | 25.0 | SSR | ATCCACCTGCTGATCAGCTC | CGAGAAGCTCAAGGTGAACC |
| RM306 | 1 | 25.5 | SSR | CAAGGTCAAGAATGCAATGG | GCCACTTTAATCATTGCATC |
| RM5461 | 1 | 26.9 | SSR | GTGGAGGAGCGAAGGGAACACG | CCTCCCATATAAACCGGCGAACC |
| RM246 | 1 | 29.9 | SSR | GAGCTCCATCAGCCATTCAG | CTGAGTGCTGCTGCGACT |
| R01D124 | 1 | 31.7 | STS | ATAGTTCGCCATCGTCAT | ACACGCCATAGCAAGGAA |
| RM128 | 1 | 32.5 | SSR | AGCTTGGGTGATTTCTTGGAAGCG | ACGACGAGGAGTCGCCGTGCAG |
| R01D144 | 1 | 38.5 | STS | AATAGAATTACTGATGAAACCTTA | GCCCGTTACCGCTTATGT |
| RM297 | 1 | 40.5 | SSR | TCTTTGGAGGCGAGCTGAG | CGAAGGGTACATCTGCTTAG |
| RM1361 | 1 | 40.8 | SSR | ATGCTTGCAGACAATCGATGC | CTCTCCGCCTAAACAACTTGTGC |
| RM5310 | 1 | 41.2 | SSR | GGGACCAAGACCTTTCCAATGC | GCGGAAGCAGGAGAATCGTAGC |
| R01D182 | 1 | 42.9 | STS | GTAATTGAATTTCACTGCTGCT | ACGTACGTGACGTGCTTATG |
| RM154 | 2 | 1.0 | SSR | ACCCTCTCCGCCTCGCCTCCTC | CTCCTCCTCCTGCGACCGCTCC |
| R02D10 | 2 | 2.6 | STS | GCACATCGGTAACGGTAGAGG | CGGTGGATAAAGACAAAGAGG |
| R02D23 | 2 | 4.6 | STS | ACCAAGATCCTGGAGGCATT | TCCAACATGGCCACAGATTA |
| R02D37 | 2 | 6.4 | STS | CCCAGTCTGCTGCCATCT | GAATGTATTTCAGTTCCAGTAAG |
| RM5699 | 2 | 9.0 | SSR | TATTGGTATGTGTGGGATCG | CTGGTGTACCTATATGGATTGC |
| RM521 | 2 | 10.5 | SSR | TTCCCTTATTCCTGCTCTCC | GGGATTTGCAGTGAGCTAGC |
| RM290 | 2 | 10.8 | SSR | ACCCTTATTCCTGCTCTCCTC | GTGCTGTAGATGGAAGGGAG |
| RM174 | 2 | 12.3 | SSR | AGCGACGCCAAGACAAGTCGGG | TCCACGTCGATCGACACGACGG |
| R02D55 | 2 | 15.2 | STS | GCAGCAAAGTGCGGAGTA | CAGGTGAATTGCCAATTT |
| RM424 | 2 | 17.1 | STS | TTTGTGGCTCACCAGTTGAG | TGGCGCATTCATGTCATC |
| R02D75 | 2 | 19.7 | STS | CCACATCCTCTCATCTCTGTCA | CGACAGGTTCAGCTTCAGGT |
| RM3688 | 2 | 22.4 | SSR | GTTGAATCAAGCTGTGCAGC | AGCTAGGCAAAGCATGCATG |
| RM6617 | 2 | 24.8 | SSR | CTCCTCCTCCCACCTCTACTCC | TAATAGATGGCGATGGACGAAGG |
| RM6465 | 2 | 28.4 | SSR | CCTAGGTTCCCATCGCAACTGACC | TTCCCGAGGAGGGAGACGAAGG |
| R02D129 | 2 | 30.3 | STS | CCTGAAGGAAATGATAGCAATAG | GTTTTGTATGCTCTTCACTTGTC |
| RM240 | 2 | 31.5 | SSR | CCTTAATGGGTAGTGTGCAC | TGTAACCATTCCTTCCATCC |
| RM106 | 2 | 32.0 | SSR | CGTCTTCATCATCGTCGCCCCG | GGCCCATCCCGTCGTGGATCTC |
| RM425 | 2 | 32.3 | SSR | ACCACAGCAGGTGGAACAGG | GCTAGCTAAGCCAACACCAACG |
| R02D144 | 2 | 33.5 | STS | AGTCCACACGCCATCGTC | TCGCAGAACTCCAAACACAC |
| RM166 | 2 | 34.3 | SSR | GGTCCTGGGTCAATAATTGGGTTACC | TTGCTGCATGATCCTAAACCGG |
| RM48 | 2 | 35.5 | SSR | TGTCCCACTGCTTTCAAGC | CGAGAATGAGGGACAAATAACC |
| RM573 | 2 | 37.3 | SSR | CCAGCCTTTGCTCCAAGTAC | TCTTCTTCCCTGGACCACAC |
| RM6349 | 3 | 0.8 | SSR | ATGATGCCTCATGTCTCTGATCTCC | AGATGAACACGACCGATAGGATAGG |
| RM569 | 3 | 1.9 | SSR | CTGCGTCAGATTTCTCCTCTTCG | ACATTCTCGCTTGCTCCTCTCG |
| RM6038 | 3 | 4.8 | SSR | CCGTCGTGTCGTACTGGTAGTCC | TCCACAGCCTACCTAGCTTCTCC |
| RM1022 | 3 | 7.2 | SSR | GTCTTTGATAGCGGCTTTGTCC | GGATGAGGGAGTAATGTCTCTTGG |
| RM545 | 3 | 9.1 | SSR | CAATGGCAGAGACCCAAAAG | CTGGCATGTAACGACAGTGG |
| RM232 | 3 | 9.7 | SSR | CCGGTATCCTTCGATATTGC | CCGACTTTTCCTCCTGACG |
| RM5955 | 3 | 11.2 | SSR | TCGCCGTAGGGCCAGTAGAAGC | CATCCACAACCTCCTGCAGTTCC |
| RM282 | 3 | 12.4 | SSR | CTGTGTCGAAAGGCTGCAC | CAGTCCTGTGTTGCAGCAAG |
| RM6929 | 3 | 12.9 | SSR | TTCTTTCGAGGGTACGTAGAGG | CTAGCTAGCCAGTAGCTGATCG |
| RM1164 | 3 | 14.7 | SSR | TTTCTGGCGACGTGATTTGTCG | CAATTCGGAAGAGCAAACATGACC |
| R03D86 | 3 | 19.7 | STS | AGGCTAAGTGAAGAAATAATAAG | CTCCGTATTCATTACTGGTTG |
| RM251 | 3 | 20.6 | SSR | GAATGGCAATGGCGCTAG | ATGCGGTTCAAGATTCGATC |
| RM16 | 3 | 22.9 | SSR | CGCTAGGGCAGCATCTAAA | AACACAGCAGGTACGCGC |
| RM563 | 3 | 23.1 | SSR | CGACCCTAGGGTTTCTCC | CTCGACGTCGTGGAAAGC |
| RM6266 | 3 | 23.6 | SSR | CACCTTCTTGAGAAGCTCCTTCG | GACATCGAGAGCGAGGACAGC |
| R03D110 | 3 | 26.3 | STS | GCATTGAATTGTACTCTTATTATAT | ACGAATCAAAAGGAGACTAAAAT |
| R03D120 | 3 | 27.4 | STS | TGGCACAAATCATTATGATC | GATTGCAATGCAGCATATAG |
| RM532 | 3 | 28.1 | SSR | TCTATAATGTAGCCCCCCCC | TTTCAGGGGCTTCTACCAAC |
| R03D143 | 3 | 32.8 | STS | AGGAGAAGCTCGCCATGAT | CATGAACTGCAACATCACCAG |
| R03D146 | 3 | 33.5 | STS | ACACTGGCTACGGCAAAG | TTTGTTCGGGAATAATGATGC |
| RM143 | 3 | 34.1 | SSR | GTCCCGAACCCTAGCCCGAGGG | AGAGGCCCTCCACATGGCGACC |
| R03D158 | 3 | 36.2 | STS | CTGCAGGGACACCGAGAT | CCACAGCGACATCCTGTACC |
| R03D159 | 3 | 36.3 | STS | CGACGGCAGATACGATACAC | CACCGCTGTTCAAAGTTTGTC |
| RM85 | 3 | 37.2 | SSR | CCAAAGATGAAACCTGGATTG | GCACAAGGTGAGCAGTCC |
| RM426 | 3 | 40.9 | SSR | ATGAGATGAGTTCAAGGCCC | AACTCTGTACCTCCATCGCC |
| RM7585 | 4 | 0.2 | SSR | TGCAATCGCGTAGTTGGTAGAATAGG | CTCGACTACCTCGCCATCATCC |
| RM3216 | 4 | 1.1 | SSR | AAGTCCGGTGACTCAGTAATCACG | GCTATATCCGGGCTCTTTCTTGC |
| RM261 | 4 | 6.5 | SSR | CTACTTCTCCCCTTGTGTCG | TGTACCATCGCCAAATCTCC |
| R04D20 | 4 | 11.6 | STS | AGTGCTCGGTTTTGTTTTC | GTCAGATATAATTGATGGATGTA |
| RM471 | 4 | 14.0 | SSR | ACGCACAAGCAGATGATGAG | GGGAGAAGACGAATGTTTGC |
| R04D35 | 4 | 18.1 | STS | GCTTCTCCTGGTTGTATGC | AAAATAGGGAGGCAGATAGAC |
| RM564 | 4 | 19.8 | SSR | CATGGCCTTGTGTATGCATC | ATGCAGAGGATTGGCTTGAG |
| R04D76 | 4 | 24.6 | STS | CTTGAACCTGAGTGAGTGG | CGATGAAAATGATGTCTA |
| RM252 | 4 | 25.7 | SSR | TTCGCTGACGTGATAGGTTG | ATGACTTGATCCCGAGAACG |
| RM3288 | 4 | 27.5 | SSR | CAATCTGGAGGCACTGTCACG | AGTGACAAGATGAAGCCAACAGC |
| R04D95 | 4 | 29.0 | STS | TTTTGTGAAACTTGACCCTC | GCGTCCATGTCTTTATTGTG |
| RM3217 | 4 | 30.3 | SSR | GACCTAACCAATCGGACGAACG | CACTAGTGCTCTGTCTCTGCTTACCC |
| RM317 | 4 | 30.8 | SSR | CATACTTACCAGTTCACCGCC | CTGGAGAGTGTCAGCTAGTTGA |
| RM348 | 4 | 32.9 | SSR | CATGAAGCTGTGTTGCTGTTGC | CGCTACTAATAGCAGAGAGACCATCG |
| R04D123 | 4 | 34.0 | STS | GAAGATTTTGCGTGTTGCTG | TCCGATAGCCCATCTGTACC |
| RM122 | 5 | 0.3 | SSR | GAGTCGATGTAATGTCATCAGTGC | GAAGGAGGTATCGCTTTGTTGGAC |
| RM413 | 5 | 2.2 | SSR | CCAATCTTGTCTTCCGGATCTTGC | AGATAGCCATGGGCGATTCTTGG |
| R05D22 | 5 | 2.6 | STS | GGGGTCGATTTCAAGTGGTA | GGAAGATGAAGACGGTGGTG |
| RM5874 | 5 | 3.5 | SSR | GAAATCCCATTTCGTTGCTGATGG | CACTCAGCATCATCGCCAGAGC |
| R05D41 | 5 | 5.9 | STS | GAGAAAGAGTGGAAGGAG | AGTATCGTCAGGAGGGTC |
| RM5994 | 5 | 6.8 | SSR | ATCAGGCACTGCAGCATGTCG | TTGAACAACTCGTCACCCTCATCG |
| RM289 | 5 | 7.8 | SSR | TTCCATGGCACACAAGCC | CTGTGCACGAACTTCCAAAG |
| RM574 | 5 | 10.7 | SSR | GGCGAATTCTTTGCACTTGG | ACGGTTTGGTAGGGTGTCAC |
| R05D55 | 5 | 13.9 | STS | CTCGCTGTTTACTGACTGG | TTTGATGTACTGCCTGCTCT |
| RM6645 | 5 | 14.9 | SSR | CTCCGGGATGCCATAGTTTCG | AAGCTTCCTCTCGATCGTCTTCG |
| RM598 | 5 | 17.8 | SSR | GAATCGCACACGTGATGAAC | ATGCGACTGATCGGTACTCC |
| RM430 | 5 | 18.6 | SSR | GTCCCTGATCAGAAACGAGATGG | TAGGGTTGGAAGAATGCAAGACC |
| R05D87 | 5 | 21.5 | STS | CTCAATTTCACCCATCCC | CGCTCCGTCTCCAACCTC |
| RM5642 | 5 | 22.1 | SSR | AAAAACCGGCTAATCCCTCC | TTCGATGGGATTGATCGC |
| RM3870 | 5 | 22.8 | SSR | GGAGTAGATGTAAAGCCAAAGGATGC | CATGTCTGAGTATGACGGAGTATTGC |
| RM3476 | 5 | 24.0 | SSR | TGATAGTTGACAATGCAGGAGAGG | TCGATCCGGAAGTTATTTCTGC |
| RM7081 | 5 | 24.7 | SSR | CTTCCCGCACTACACTGCACTCC | CTGCAACTTGCTCATGGAGTTGG |
| RM3348 | 5 | 25.2 | SSR | CTTCTCGGTTCATCCAAAGAGC | GTGGAAGCTATGGGTAGCTCACG |
| RM6972 | 5 | 25.5 | SSR | CATGGTGCTCCTACTGGTTGTACC | CCCATCCATAATCACAACTCAGC |
| RM3321 | 5 | 25.9 | SSR | CTATAAATAGGGCCAGGTGGTAGGC | CTGCCTAGCCATAGCCAAACG |
| RM3616 | 5 | 26.4 | SSR | ACACCAGCGACGATCGATTCC | CTGCGTGAGACGGTTGACTGACC |
| RM6360 | 5 | 28.3 | SSR | ACGTGGAATCCAAATTGACAGC | TTCGCTGCACTGTTTACTCTTGG |
| RM334 | 5 | 28.6 | SSR | GTTCAGTGTTCAGTGCCACC | GACTTTGATCTTTGGTGGACG |
| RM421 | 5 | 28.9 | SSR | AGCTCAGGTGAAACATCCAC | ATCCAGAATCCATTGACCCC |
| RM538 | 5 | 34.5 | SSR | GGTCGTTGAAGCTTACCAGC | ACAAGCTCTCAAAACTCGCC |
| RM170 | 6 | 1.3 | SSR | TCGCGCTTCTTCCTCGTCGACG | CCCGCTTGCAGAGGAAGCAGCC |
| RM510 | 6 | 2.8 | SSR | AACCGGATTAGTTTCTCGCC | TGAGGACGACGAGCAGATTC |
| RM3805 | 6 | 2.9 | SSR | ACACCACCATCAACGTACCAACC | AAGTCGAGAGGAAGAAGCCAAGG |
| RM585 | 6 | 3.1 | SSR | CAGTCTTGCTCCGTTTGTTG | CTGTGACTGACTTGGTCATAGG |
| RM6773 | 6 | 4.4 | SSR | GCTGCTCCACCTTCACCTTCC | CGATGGTGTGTTGTTTGGTTGC |
| RM50 | 6 | 6.3 | SSR | ACTGTACCGGTCGAAGACG | AAATTCCACGTCAGCCTCC |
| RM539 | 6 | 7.9 | SSR | GAGCGTCCTTGTTAAAACCG | AGTAGGGTATCACGCATCCG |
| R06D51 | 6 | 8.1 | STS | GGCAACCGACCATCACTTAG | CCCCACTGGTCAAATGAGTT |
| RM3183 | 6 | 12.3 | SSR | GTGGTGCTAGTATGGACGAGAGG | CGGTTGGTAGACTGTAAACAAAGTGC |
| RM136 | 6 | 13.3 | SSR | GAGAGCTCAGCTGCTGCCTCTAGC | GAGGAGCGCCACGGTGTACGCC |
| RM527 | 6 | 15.9 | SSR | GGCTCGATCTAGAAAATCCG | TTGCACAGGTTGCGATAGAG |
| RM541 | 6 | 19.6 | SSR | TATAACCGACCTCAGTGCCC | CCTTACTCCCATGCCATGAG |
| RM1340 | 6 | 23.0 | SSR | ATCGATCTCCACCACTTCCTTCC | CCCTACTCCCAGTAACCCAAATAGG |
| RM7434 | 6 | 23.6 | SSR | AGGCTTCTTGGAATGGAACTGC | GGGAATATACGTGGATGTGAGAGG |
| RM162 | 6 | 24.0 | SSR | GCCAGCAAAACCAGGGATCCGG | CAAGGTCTTGTGCGGCTTGCGG |
| RM6395 | 6 | 25.6 | SSR | GGCTTCGGCTTCTGAACTAGC | CGACTAAGCAGCAGTAACAATCTCG |
| RM1370 | 6 | 28.1 | SSR | AAACGAGAACCAACCGACAC | GGAGGGAGGAATGGGTACAC |
| RM340 | 6 | 28.2 | SSR | GGTAAATGGACAATCCTATGGC | GACAAATATAAGGGCAGTGTGC |
| RM439 | 6 | 29.1 | SSR | CTGGGTCTAATCTCGTCCTAAATTGC | CGCCTCTCATAACAGTCCACTCC |
| RM5814 | 6 | 29.4 | SSR | GATCTCCACCACCTCCATCTCC | CCTACATCAAGGCTCGCTACTGC |
| RM345 | 6 | 37.8 | SSR | ATTGGTAGCTCAATGCAAGC | GTGCAACAACCCCACATG |
| RM5344 | 7 | 1.9 | SSR | ATGACCTTCCTCGCAAGAGTGC | ACACAAGGCTCTCAACCAAGACG |
| RM427 | 7 | 2.7 | SSR | TTGAGCTGATGAGAGTTGGTTGC | CTGTCACTAGCTCTGCCCTGACC |
| R07D25 | 7 | 3.1 | STS | CCTGGGACCTTGTACCCTCT | TGACACCATCAGAAAACTGCT |
| R07D44 | 7 | 6.7 | STS | ACCTTCCCTCCCCTTTTGAT | AACTTGGTCTTCCTGTTTTATTG |
| RM501 | 7 | 8.7 | SSR | GCCCAATTAATGTACAGGCG | ATATCGTTTAGCCGTGCTGC |
| RM3755 | 7 | 14.7 | SSR | TGTGGACAACCTCAACTGAAAGC | CATAATCACCAACATCGGAGAAGC |
| RM351 | 7 | 16.5 | SSR | CCATCCTCCACCGCCTCTCG | TGGAGGAAGGAAAGGGGACG |
| R07D68 | 7 | 19.4 | STS | ATGTCGCCTACGAGTTTTC | TTCATGTGACCATTTGTGC |
| RM234 | 7 | 22.9 | SSR | ACAGTATCCAAGGCCCTGG | CACGTGAGACAAAGACGGAG |
| R07D82 | 7 | 23.6 | STS | CAGCCCTAAATCTAAATACCC | ACGTTGAGACAGGCGAGC |
| R07D99 | 7 | 26.4 | STS | CCGGTGACTTCTTCATGTCC | GCACACTCATCGGTGCATAC |
| R07D100 | 7 | 26.7 | STS | TGTTTTTGTTGCTCCCAGTG | GGGAGAACCTTCCCACTGAT |
| RM172 | 7 | 29.5 | SSR | TGCAGCTGCGCCACAGCCATAG | CAACCACGACACCGCCGTGTTG |
| RM248 | 7 | 30.3 | SSR | TCCTTGTGAAATCTGGTCCC | GTAGCCTAGCATGGTGCATG |
| RM3710 | 8 | 0.4 | SSR | AGCAGCAGCCGCTTCTTGTCG | CGATTGTTTCCTCCGCCATTCC |
| RM6393 | 8 | 0.6 | SSR | TCTGGATGTAGTCGATGCTGAGG | AGAAGAGAGCAGCGTGACATGG |
| RM152 | 8 | 0.6 | SSR | GAAACCACCACACCTCACCG | CCGTAGACCTTCTTGAAGTAG |
| R08D17 | 8 | 2.1 | STS | TCTGGACTGGAAATAGCATGG | GTGTGAAAGCCCATCTGTCA |
| RM1376 | 8 | 3.2 | SSR | ATGCATGTGTGATGACTGACAGG | GGTACTCTTGCCAAATGGTCTCC |
| RM3572 | 8 | 3.9 | SSR | CCATTTGGTAGGTCCATCTTACCC | CTCCCAAGTGAAGTGCTGTCTGG |
| RM3181 | 8 | 7.5 | SSR | TTTAGCGAAGCAAACCCTCACC | CGAGCCAAGAAGAATGAATCAGC |
| RM8271 | 8 | 7.6 | SSR | AGCAGCTCCGATTGTGTTAGCC | AATGGCGTCTGTGGTACTTTGC |
| R08D50 | 8 | 8.8 | STS | GGAGGAGGAGGAGAGATGGT | GCAGCAGAACATGAGTTTGG |
| R08D54 | 8 | 13.4 | STS | CCTATTCACTCTACCGACAT | GTTTAGTTCCCATTGCTTT |
| RM310 | 8 | 14.8 | SSR | CCAAAACATTTAAAATATCATG | GCTTGTTGGTCATTACCATTC |
| RM331 | 8 | 17.9 | SSR | GAACCAGAGGACAAAAATGC | CATCATACATTTGCAGCCAG |
| RM223 | 8 | 20.6 | SSR | GAGTGAGCTTGGGCTGAAAC | GAAGGCAAGTCTTGGCACTG |
| R08D77 | 8 | 20.7 | STS | CGAAAGAGGAGAGGGGTAGT | CGAAAACGAGAAACAAATA |
| RM284 | 8 | 21.1 | SSR | ACTGCATGATCCTCCTCAGATCC | CCCTCTGATCTCTGATACTCCATCC |
| RM3262 | 8 | 22.4 | SSR | CTGGAGATGCAGATCCTCAACC | TAGTACAACATGGGAGCCTGTCG |
| RM6976 | 8 | 23.6 | SSR | CTGCAACCTGCACGAGTACACC | GTCCCATTGGATAGAATCCCAGAGC |
| RM230 | 8 | 25.8 | SSR | GCCAGACCGTGGATGTTC | CACCGCAGTCACTTTTCAAG |
| RM7400 | 8 | 27.3 | SSR | TTTGATTTGTGCAGGGATACGC | CTGCAGCAGAAACACGAAGAGG |
| RM264 | 8 | 27.9 | SSR | GTTGCGTCCTACTGCTACTTC | GATCCGTGTCGATGATTAGC |
| RM433 | 8 | 30.2 | SSR | TGCGCTGAACTAAACACAGC | AGACAAACCTGGCCATTCAC |
| RM264 | 8 | 33.4 | SSR | GTTGCGTCCTACTGCTACTTC | GATCCGTGTCGATGATTAGC |
| RM316 | 9 | 1.0 | SSR | CTAGTTGGGCATACGATGGC | ACGCTTATATGTTACGTCAAC |
| RM321 | 9 | 8.3 | SSR | CCAACACTGCCACTCTGTTC | GAGGATGGACACCTTGATCG |
| R09D28 | 9 | 9.4 | STS | ACTGCTTTGATGGCTTGTG | CTCCCCAAACTGAATCC |
| RM409 | 9 | 11.9 | SSR | CCGTCTCTTGCTAGGGATTC | GGGGTGTTTTGCTTTCTCTG |
| RM105 | 9 | 12.5 | SSR | GTCGTCGACCCATCGGAGCCAC | TGGTCGAGGTGGGGATCGGGTC |
| R09D51 | 9 | 14.7 | STS | CTCACCTACCTAAAACCCAAC | CCACCCAAATCTGATACTG |
| RM3700 | 9 | 15.4 | SSR | CCTTTGCCGCCTTCTCTTGG | ACGAGTTCCCGGTTAACCTTACG |
| RM257 | 9 | 17.7 | SSR | CCGTGCAACTTAAATCCAAACAGG | GGAATCCTATATGAGCCAGTGATGG |
| R09D75 | 9 | 19.2 | STS | CTATAAGACCAAAACGAAAACT | GAAAACCATTGTGTCACTGTA |
| RM107 | 9 | 19.8 | SSR | TCTTACTGCGTCCTCTGGGTTCC | ATTCTTGCGGCGATTCATCTTCC |
| RM215 | 9 | 20.9 | SSR | GAGCAGCAAGAGCAGCAGAGG | CATGCTCGACTTCAGAAGCTTGG |
| R09D92 | 9 | 22.5 | STS | GACAGAGAAACTGGGCGTTC | CTGTTAAACCCCCACAAAGC |
| RM205 | 9 | 22.7 | SSR | CTGGTTCTGTATGGGAGCAG | CTGGCCCTTCACGTTTCAGTG |
| RM189 | 9 | 23.6 | SSR | CGTCTTCCCCAACGCTAAAA | CGCCGGGCTTCGCTTC |
| RM245 | 9 | 29.2 | SSR | ATGCCGCCAGTGAATAGC | CTGAGAATCCAATTATCTGGGG |
| RM6370 | 10 | 0.3 | SSR | TTGACAAGCCACACACACAG | GTCCTCCCTTGGTTCTTTCC |
| RM216 | 10 | 4.6 | SSR | GCATGGCCGATGGTAAAG | TGTATAAAACCACACGGCCA |
| R10D16 | 10 | 8.4 | STS | AATGCCACATCTTCCTTCAAA | CAGGACAGAGGAGAGGGAGA |
| RM239 | 10 | 9.7 | SSR | TACAAAATGCTGGGTACCCC | ACATATGGGACCCACCTGTC |
| RM596 | 10 | 14.2 | SSR | ATCTACACGGACGAATTGCC | AGAAGCTTCAGCCTCTGCAG |
| R10D38 | 10 | 16.0 | STS | TAAGACCTTTGCCTGCTGGT | GAGCAGATTTTTCCCACTGC |
| R10D45 | 10 | 17.4 | STS | CAAGCCTGGATCACCATCTC | CTCCACAGCGGTAAGTGTTG |
| R10D57 | 10 | 19.4 | STS | GTCCCTAGGCCATCTCTTG | GCGAATAGGGGTGGACAG |
| R10D61 | 10 | 20.3 | STS | TGGGGAACTGTTGAAGATGA | ATCGTGGGCAAAGCAATACT |
| RM1374 | 10 | 21.6 | SSR | TAGATATGTTGGGCCGGAAG | AGATCGATGCCGTTTCAGAC |
| RM591 | 10 | 22.5 | SSR | CTAGCTAGCTGGCACCAGTG | TGGAGTCCGTGTTGTAGTCG |
| RM6824 | 10 | 23.1 | SSR | GAGAGAACCTGGTGGTGGAG | AGTGGTAGAAGATCCGAGATCG |
| RM484 | 10 | 25.3 | SSR | TCTCCCTCCTCACCATTGTC | TGCTGCCCTCTCTCTCTCTC |
| RM6395 | 10 | 25.6 | SSR | GGCTTCGGCTTCTGAACTAGC | CGACTAAGCAGCAGTAACAATCTCG |
| RM591 | 10 | 30.8 | SSR | CTAGCTAGCTGGCACCAGTG | TGGAGTCCGTGTTGTAGTCG |
| RM7557 | 11 | 2.3 | SSR | CCTCCAGGTGAAGTGCCTTTGC | CTTCATCTTCCCTGCTGCCTTTGC |
| RM1124 | 11 | 3.8 | SSR | CTAGGGATCGGTAGACCCAATCG | TGGTGATGGCACTTTAGACAGAAGG |
| R11D24 | 11 | 4.3 | STS | TTCGGCATCTACCATGACTG | CTTGATCTCGTCCACCGTCT |
| RM120 | 11 | 5.7 | SSR | CACACAAGCCCTGTCTCACGACC | CGCTGCGTCATGAGTATGTA |
| RM3701 | 11 | 8.0 | SSR | GAAAGAGGAGGAAGAGCTAGAGG | CCATATGTACGGAGTGTGTTTACC |
| R11D45 | 11 | 8.1 | STS | AGTTTTGACTGATAGCCGATTG | GAGAAGAAAGAGGAGGAAGAGC |
| RM441 | 11 | 11.4 | SSR | ACACCAGAGAGAGAGAGAGAGAG | TCTGCAACGGCTGATAGATG |
| RM3428 | 11 | 13.4 | SSR | GCCATTGACACCAAATGATCACC | GGCATATAAGGTCCATGGTGAATTGG |
| RM6272 | 11 | 16.4 | SSR | ATCTACTCCGCCACCACCACAGC | CTCTACCTCTCCCTCTCGCCTTCC |
| RM287 | 11 | 16.6 | SSR | TTCCCTGTTAAGAGAGAAATC | GTGTATTTGGTGAAAGCAAC |
| RM5349 | 11 | 19.0 | SSR | CATCCAAATGTTGCGGATTACC | TTCAATAGCCCAGAGAACCAAGC |
| R11D80 | 11 | 19.9 | STS | AGGAGATACAGGGCCAGGTT | CCCTTCAGGATCAAATGGAG |
| RM457 | 11 | 21.6 | ssr | CTCCAGCATGGCCTTTCTAC | ACCTGATGGTCAAAGATGGG |
| RM187 | 11 | 22.3 | SSR | CCAAGGGAAAGATGCGACAATTG | GTGGACGCTTTATATTATGGG |
| RM254 | 11 | 24.2 | SSR | AGCCCCGAATAAATCCACCT | CTGGAGGAGCATTTGGTAGC |
| RM7170 | 11 | 24.4 | SSR | GCGACTTGAGAGCGTTTGTAGG | AGCCAACTGTAGCACGAACTGC |
| R11D100 | 11 | 24.8 | STS | AAGAAAAATATCTATTGAGGAGTG | GGAGGACCATAAATGACGG |
| R11D110 | 11 | 26.5 | STS | GCGGAAAAGCAAGAGGAAT | GCCCATGAGAGAAGTAAAGGAA |
| RM2064 | 11 | 26.6 | SSR | TTGATGTTGTGGGCTTCTCTACC | TCCAATCACGCGTATTACATGC |
| RM206 | 11 | 26.8 | SSR | CCCATGCGTTTAACTATTCT | CGTTCCATCGATCCGTATGG |
| RM139 | 11 | 31.5 | SSR | GAGAGGGAGGAAGGGAGGCGGC | CTGCCATGGCAGAGAAGGGGCC |
| RM4A | 12 | 0.9 | SSR | TTGACGAGGTCAGCACTGAC | AGGGTGTATCCGACTCATCG |
| RM8216 | 12 | 1.4 | SSR | AGTTGATTATCCATCATGTGC | TGTAGGCCCTATGAATCTCC |
| RM6296 | 12 | 3.2 | SSR | TTAAGCCCACGTTTCTCTTGTCC | CTCGCTAGGGTTAGGGTTTCAGG |
| RM7619 | 12 | 4.8 | SSR | TCTTGGTATGTATTGGCAGCGAAAGC | AGGATGTGAATGAAGGCGAATGG |
| RM7003 | 12 | 6.8 | SSR | CTCTAGCTCTCTCATGGATGG | AATCATAGGGCAGACATACAGC |
| RM453 | 12 | 7.3 | SSR | CGCATCTCTCTCCCTTATCG | CTCTCCTCCTCGTTGTCGTC |
| R12D51 | 12 | 11.4 | STS | CCTAACAGCGGCATCTCCT | AGTGCATTCACCCAAAAATTG |
| RM511 | 12 | 15.5 | SSR | CTTCGATCCGGTGACGAC | AACGAAAGCGAAGCTGTCTC |
| RM519 | 12 | 16.3 | SSR | AGAGAGCCCCTAAATTTCCG | AGGTACGCTCACCTGTGGAC |
| RM277 | 12 | 18.3 | SSR | CGGTCAAATCATCACCTGAC | CAAGGCTTGCAAGGGAAG |
| RM1246 | 12 | 19.2 | SSR | GGCTCACCTCGTTCTCGATCC | CATAAATAAATAGGGCGCCACACC |
| R12D70 | 12 | 19.8 | STS | TTGATGATAGTATTTGCTGATG | AGATAGTGTCGGCGGTGG |
| RM235 | 12 | 23.7 | SSR | AGAAGCTAGGGCTAACGAAC | TCACCTGGTCAGCCTCTTTC |
| RM6947 | 12 | 24.0 | SSR | GGCTGCTGGTATGTATTCAAGTGC | GCAAGTGAGAAACAGAAGTGATCG |
| R12D103 | 12 | 26.0 | STS | CCGCCGAGAAGAAACAAAG | CCCAAGAACAGGATTACA |
| RM7558 | 12 | 27.0 | SSR | CAGTAGCAGGCTCCCTTTTG | ATCAGGAACACCAGAGACGG |
| RM1227 | 12 | 27.4 | SSR | CATCGACATGTGGACCACTCC | GCCTGAGACAAGTCCATGGTAGC |
