# Supplementary material for: Gene Mapping, Genome-Wide Transcriptome Analysis, and WGCNA Reveals the Molecular Mechanism for Triggering Programmed Cell Death in Rice Mutant pir1
Source: Plants (Basel). 2020 Nov 19;9(11):1607. doi: 10.3390/plants9111607 (PMC7699392; doi:10.3390/plants9111607)
Supplement: Supplementary file 1 [file plants-09-01607-s001.zip › Supplementary files/Table S35.docx]

**Table S35.** Primers are listed for real-time PCR.

| Gene ID | Description | Primer sequence (From 5′ to 3′) |
| --- | --- | --- |
| LOC4335223 | Probable cinnamyl alcohol dehydrogenase 6 | F: GGAGCATGACGGGTGGGATG  R: ATTCTGCGTGCAGAGCCTCC |
| LOC4348242 | Probable cinnamyl alcohol dehydrogenase 1 | F: GGCTTGGGTCACATGGCAGT  R:GAATGGGTGATCGCCGGAGG |
| LOC4336415 | Phenylalanine Ammonia-Lyase | F: CGACCAAACTCTGTGGCGGT  R: TCTGTCCCGGGTGGTGCTTA |
| LOC4345689 | Cinnamoyl-CoA reductase 1 | F: GAGCTGGTGGTGGTGAACCC  R:CACCTGGTGGGGACAGGGTA |
| LOC4331650 | 4-coumarate--CoA ligase-like 1 | F: GCCCAACGTGTTCCTCTGCT  R: CTCTGCACGGCCTCCATCAG |
| LOC4332121 | Allene oxide synthase 2-like | F: GTCGAATGGGCGAGAGACGG  R: GCAGTACGATTGACGGCGGA |
| LOC4337904 | Acyl-coenzyme A oxidase 4, peroxisomal | F: CTTGGCCGTGAGCTACTGGG  R: GAAGCTGGCGATTCCGGTGA |
| LOC4340986 | Acyl-coenzyme A oxidase 3, peroxisomal | F: TCCGGACTACAACGAGGGCA  R: TGATGGCGAGGGAGTGGTCA |
| LOC4327329 | Cytochrome P450 90D2 | F: ACCGACGAGCTCATCTCCGA  R: GCGCATGATCCCACCGATGA |
| LOC4349746 | Cytochrome P450 90A3-like | F: CTCTCGCCAACCTCCTCCCT  R:GCCACCAGCAGAGAAAGGCA |
| LOC4351461 | Cytochrome P450 90A4-like | F:GAGGAAGAAGGTGGCCGGTG  R: GCGTCATGAGCACGGACGTA |
| LOC4350584 | Leucine-Rich Repeat Protein 2 | F: TACCAGCAGCACTGGGCAAC  R: GCAGGGATGGTGCCACTCAA |
| LOC4332405 | Receptor-Like Cytoplasmic Kinase 176 | F: ACAGGCCATTTGACCGCGAA  R: GGCGTGAGTCGAGGACATGG |
| LOC4339239 | Universal Stress Protein PHOS32 | F: ACCATGAAGCTCCCGAGGGT  R: GGCATTAGCAAGTCGCACGC |
| LOC4337287 | Protein HEAT INTOLERANT 4 | F: TCCCCCAAATCGCACACACC  R: GACGCGCCATCTTCTCCCTC |
| LOC4344706 | Reticuline oxidase | F: GAAATTTGGGCTCGCCGCTG  R: GACGTTGTGAGGAACCGGCA |
| LOC4341560 | Cinnamoyl-CoA reductase 1 | F: CGACGGGAGAACGAAGACGG  R: ATCAGGTGGGCGTTCTTGGC |
| LOC9270429 | Cinnamoyl-CoA reductase 1-like | F: AGCGGCAAAGTTCAACCGGA  R: AGCTGCAGATTCTCCGTGGC |
| LOC4336750 | 1-aminocyclopropane-1-carboxylate synthase | F: TCCGATCATCCTTGCGTGGC  R: CGATGCCCTGGTACGCCATT |
| LOC107279225 | 2-oxoglutarate-Fe(II) type oxidoreductase hxnY-like | F: GCTGGAAGCCGGAGATGGTC  R: AGCGGCTGCTCGAAAAACCT |
| LOC4340667 | 1-aminocyclopropane-1-carboxylate oxidase homolog 4 | F: CATCGCCATCAACAACGCGG  R: GAAGATGACCGGGACGGCAG |
| LOC4336987 | Lecithin-cholesterol acyltransferase-like 4 | F: CACTGCCCATACCGTTCGCT  R: ACTGCATCGAGGCCATCTGC |
| LOC4331515 | 4-coumarate--CoA ligase-like 4 | F: GTTCCACGTCTACGGCCTCG  R: GCACGAGCGGAAGGTAGGTC |
| LOC4328997 | Peroxisomal fatty acid beta-oxidation multifunctional protein-like | F: TGTCACGCCTTTGGGCTCTG  R: CATCTAGGCAGGCCTGGTGC |
| LOC4333823 | Probable linoleate 9S-lipoxygenase 4 | F: GCACTACCGCGACACGATGA  R: GAGATCGACGGGGAGAGCCT |
| LOC4340486 | putative 12-oxophytodienoate reductase 4 | F: GATCACCCATGGCCTCCTGC  R: TGATCGCGAACCTCTTGGGC |
| LOC4345762 | 12-oxophytodienoate reductase 7-like | F: CATTCATGTGCAGTGGCGGC  R: GTACTTGTTCAGCCCGGCGT |
| LOC4350881 | acyl-coenzyme A oxidase 2, peroxisomal | F: GTGCGACCTTTACGCGCTTG  R: AGACTGCATGCCGATTGGGG |
|  | *Actin* | F:GAGTATGATGAGTCGGGGTCCAG  R: ACACCAACAATCCCAAACAGAG |
