# Supplementary material for: Gene Mapping, Genome-Wide Transcriptome Analysis, and WGCNA Reveals the Molecular Mechanism for Triggering Programmed Cell Death in Rice Mutant pir1
Source: Plants (Basel). 2020 Nov 19;9(11):1607. doi: 10.3390/plants9111607 (PMC7699392; doi:10.3390/plants9111607)
Supplement: Supplementary file 1 [file plants-09-01607-s001.zip › Supplementary files/Table S4.docx]

**Table S4**. Overview of the RNA-sequencing reads generated from each sample.

| Sample | Raw Reads_count | Clean Reads_count (%) | Total Mapped Reads_count | Mapped% | Uniquely Mapped Reads_count | Uniquely Mapped% |
| --- | --- | --- | --- | --- | --- | --- |
| ZJ22a1 | 53433648 | 53326050(99.80%) | 49658030 | 97.08% | 48639509 | 95.09% |
| ZJ22a2 | 52123334 | 52001596(99.77%) | 47997298 | 96.95% | 46953816 | 94.84% |
| ZJ22a3 | 56472144 | 56344398(99.77%) | 51424674 | 96.81% | 50292740 | 94.68% |
| ZJ22b1 | 49380638 | 49266546(99.77%) | 44991431 | 96.80% | 43983020 | 94.63% |
| ZJ22b2 | 56126380 | 56002526(99.78%) | 51376248 | 96.89% | 50185506 | 94.64% |
| ZJ22b3 | 55990500 | 55869032(99.78%) | 51667713 | 96.94% | 50460104 | 94.67% |
| ZJ22c1 | 52776016 | 52663698(99.79%) | 48743765 | 96.88% | 47648573 | 94.70% |
| ZJ22c2 | 49130382 | 49019806(99.77%) | 45244576 | 96.75% | 44214973 | 94.55% |
| ZJ22c3 | 55495632 | 55376364(99.79%) | 51737399 | 96.82% | 50544501 | 94.59% |
| *pir1*a1 | 46220796 | 46117486(99.78%) | 40951439 | 97.13% | 40070876 | 95.04% |
| *pir1*a2 | 51078126 | 50977136(99.80%) | 43483134 | 97.20% | 42595431 | 95.22% |
| *pir1*a3 | 48979764 | 48877618(99.79%) | 44360523 | 97.04% | 43430487 | 95.01% |
| *pir1*b1 | 47571022 | 47460282(99.77%) | 44427983 | 97.04% | 43450144 | 94.90% |
| *pir1*b2 | 42144316 | 42050830(99.78%) | 38242719 | 96.62% | 37429266 | 94.56% |
| *pir1*b3 | 53022674 | 52902422(99.77%) | 49532806 | 97.06% | 48419946 | 94.88% |
| *pir1*c1 | 45305708 | 45207812(99.78%) | 42479868 | 96.98% | 41536505 | 94.83% |
| *pir1*c2 | 54290492 | 54176626(99.79%) | 49904566 | 96.26% | 48799444 | 94.13% |
| *pir1*c3 | 55247654 | 55121558(99.77%) | 51718421 | 96.76% | 50522786 | 94.53% |
